# Chromosome contacts in activated T cells identify autoimmune disease candidate genes

**DOI:** 10.1186/s13059-017-1285-0

**Published:** 2017-09-04

**Authors:** Oliver S. Burren, Arcadio Rubio García, Biola-Maria Javierre, Daniel B. Rainbow, Jonathan Cairns, Nicholas J. Cooper, John J. Lambourne, Ellen Schofield, Xaquin Castro Dopico, Ricardo C. Ferreira, Richard Coulson, Frances Burden, Sophia P. Rowlston, Kate Downes, Steven W. Wingett, Mattia Frontini, Willem H. Ouwehand, Peter Fraser, Mikhail Spivakov, John A. Todd, Linda S. Wicker, Antony J. Cutler, Chris Wallace

**Affiliations:** 1 Department of Medicine, University of Cambridge, Addenbrooke’s Hospital, Cambridge, CB2 0SP UK; 2 JDRF/Wellcome Trust Diabetes and Inflammation Laboratory, Department of Medical Genetics, NIHR Cambridge Biomedical Research Centre, Cambridge Institute for Medical Research, University of Cambridge, Cambridge, CB2 0XY UK; 3 Present address: JDRF/Wellcome Trust Diabetes and Inflammation Laboratory, Wellcome Trust Centre for Human Genetics, Nuffield Department of Medicine, NIHR Oxford Biomedical Research Centre, University of Oxford, Roosevelt Drive, Oxford, OX3 7BN UK; 4 Nuclear Dynamics Programme, The Babraham Institute, Babraham Research Campus, Cambridge, CB22 3AT UK; 5 Department of Haematology, University of Cambridge, Cambridge Biomedical Campus, Long Road, Cambridge, CB2 0PT UK; 6 National Health Service Blood and Transplant, Cambridge Biomedical Campus, Long Road, Cambridge, CB2 0PT UK; 7 British Heart Foundation Centre of Excellence, Division of Cardiovascular Medicine, Addenbrooke’s Hospital, Hills Road, Cambridge, CB2 0QQ UK; 8 Department of Human Genetics, Wellcome Trust Sanger Institute, Wellcome Trust Genome Campus, Hinxton, Cambridge, CB10 1HH UK; 9 MRC Biostatistics Unit, University of Cambridge, Cambridge Institute of Public Health, Cambridge Biomedical Campus, Cambridge, CB2 0SR UK

**Keywords:** Genetics, Genomics, Chromatin conformation, CD4^+^ T cells, CD4^+^ T cell activation, Autoimmune disease, Genome-wide association studies

## Abstract

**Background:**

Autoimmune disease-associated variants are preferentially found in regulatory regions in immune cells, particularly CD4^+^ T cells. Linking such regulatory regions to gene promoters in disease-relevant cell contexts facilitates identification of candidate disease genes.

**Results:**

Within 4 h, activation of CD4^+^ T cells invokes changes in histone modifications and enhancer RNA transcription that correspond to altered expression of the interacting genes identified by promoter capture Hi-C. By integrating promoter capture Hi-C data with genetic associations for five autoimmune diseases, we prioritised 245 candidate genes with a median distance from peak signal to prioritised gene of 153 kb. Just under half (108/245) prioritised genes related to activation-sensitive interactions. This included *IL2RA*, where allele-specific expression analyses were consistent with its interaction-mediated regulation, illustrating the utility of the approach.

**Conclusions:**

Our systematic experimental framework offers an alternative approach to candidate causal gene identification for variants with cell state-specific functional effects, with achievable sample sizes.

**Electronic supplementary material:**

The online version of this article (doi:10.1186/s13059-017-1285-0) contains supplementary material, which is available to authorized users.

## Background

Genome-wide association studies (GWAS) in the last decade have associated 324 distinct genomic regions to at least one and often several autoimmune diseases (https://www.immunobase.org). The majority of associated variants lie outside genes [1] and presumably tag regulatory variants acting on nearby or more distant genes [2, 3]. Progress from GWAS discovery to biological interpretation has been hampered by lack of systematic methods to define the gene(s) regulated by a given variant. The use of Hi-C [4] and capture Hi-C to link GWAS identified variants to their target genes for breast cancer [5] and autoimmune diseases [6] using cell lines, has highlighted the potential for mapping long-range interactions in advancing our understanding of disease association. The observed cell specificity of these interactions indicates a need to study primary disease-relevant human cells and investigate the extent to which cell state may affect inference.

Integration of GWAS signals with cell-specific chromatin marks has highlighted the role of regulatory variation in immune cells [7], and in particular CD4^+^ T cells, in autoimmune diseases [8]. Concordantly, differences in DNA methylation of immune-related genes have been observed in CD4^+^ T cells from autoimmune disease patients compared to healthy controls [9, 10]. CD4^+^ T cells are at the centre of the adaptive immune system and exquisite control of activation is required to guide CD4^+^ T cell fate through selection, expansion and differentiation into one of a number of specialised subsets. Additionally, the prominence of variants in physical proximity to genes associated with T cell regulation in autoimmune disease GWAS and the association of human leukocyte antigen haplotypes have suggested that control of T cell activation is a key etiological pathway in development of many autoimmune diseases [11].

We explored the effect of activation on CD4^+^ T cell gene expression, chromatin states and chromosome conformation. Promoter capture Hi-C (PCHi-C) was used to map promoter interacting regions (PIRs) and to relate activation-induced changes in gene expression to changes in chromosome conformation and transcription of (PCHi-C) linked enhancer RNAs (eRNAs). We also fine-mapped the most probable causal variants for five autoimmune diseases, autoimmune thyroid disease (ATD), coeliac disease (CEL), rheumatoid arthritis (RA), systemic lupus erythematosus (SLE) and type 1 diabetes (T1D). We integrated these sources of information to derive a systematic prioritisation of candidate autoimmune disease genes.

## Results

### A time-course expression profile of early CD4^+^ T cell activation

**Fig. 1:**
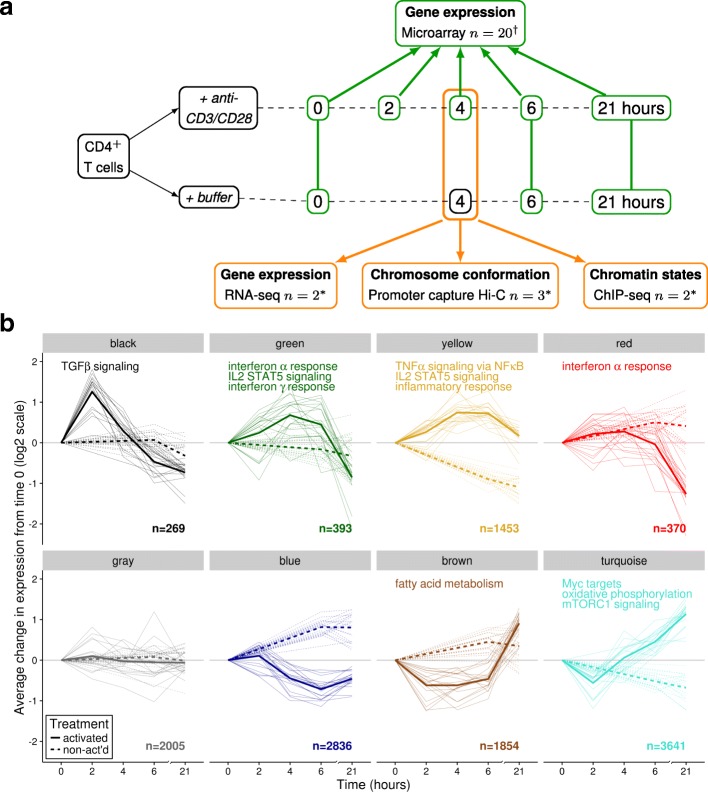
**a** Summary of genomic profiling of CD4^+^ T cells during activation with anti-CD3/CD28 beads. We examined gene expression using microarray in activated and non-activated CD4^+^ T cells across 21 h and assayed cells in more detail at the 4-h time point using ChIP-seq, RNA-seq and PCHi-C. n gives the number of individuals† or pools* assayed. **b** Eight modules of co-regulated genes were identified and eigengenes are plotted for each individual (*solid lines* = activated, *dashed lines* = non-activated), with heavy lines showing the average eigengene across individuals. We characterised these modules by gene set enrichment analysis within the MSigDB HALLMARK gene sets; where significant gene sets were found, up to three shown per module. *n* is the number of genes in each module

We profiled gene expression in CD4^+^ T cells from 20 healthy individuals across a 21 hour (h) activation time-course and identified eight distinct gene modules by clustering these profiles (Fig. 1, Additional file 1: Table S1). This experimental approach focused on much earlier events than previous large time-course studies (e.g. 6 h–8 days [12]) and highlights the earliest changes that are either not seen after or are returning towards baseline by 6 h (Additional file 2: Figure S1). Gene set enrichment analysis using MSigDB Hallmark gene sets [13] demonstrated that these modules captured temporally distinct aspects of CD4^+^ T cell activation. For example, negative regulators of TGF-beta signalling were rapidly upregulated and returned to baseline by 4 h. Interferon responses, inflammatory responses and IL-2 and STAT5 signalling pathways showed a more sustained upregulation beyond 6 h, while fatty acid metabolism was initially downregulated in favour of oxidative phosphorylation.

### PCHi-C captures dynamic enhancer-promoter interactions

We examined activated and non-activated CD4^+^ T cells purified from healthy individuals in more detail at the 4-h time point, at which the average fold change of genes related to cytokine signalling and inflammatory response was maximal, using total RNA sequencing (RNA-seq), histone mark chromatin immunoprecipitation sequencing (ChIP-seq), and PCHi-C. Of 8856 genes identified as expressed (see ‘Methods’) in either condition (non-activated or activated), 25% were upregulated or downregulated (1235 and 952 genes, respectively, false discovery rate (FDR) < 0.01, Additional file 3: Table S2). We used PCHi-C to characterise promoter interactions in activated and non-activated CD4^+^ T cells. Our capture design baited 22,076 *Hind*III fragments (median length, 4 kb) which contained the promoters of 29,131 annotated genes, 18,202 of which are protein-coding (Additional file 4: Table S3). We detected 283,657 unique PCHi-C interactions with the CHiCAGO pipeline [14], with 55% found in both activation states and 22% and 23% found only in non-activated and only in activated CD4^+^ T cells, respectively (Additional file 5: Table S4). Of the baited promoter fragments, 11,817 were involved in at least one interaction, with a median distance between interacting fragments of 324 kb. Each interacting promoter fragment connected to a median of eight PIRs, while each interacting PIR was connected to a median of one promoter fragment (Additional file 2: Figure S2).

We compared our interaction calls to an earlier ChIA-PET dataset from non-activated CD4^+^ T cells [15] and found we replicated over 50% of the longer-range interactions (100 kb or greater), with replication rates over tenfold greater for interactions found in non-activated CD4^+^ T cells compared to interactions found only in erythroblasts or megakaryocytes (Additional file 2: Figure S3). We also compared histone modification profiles in interacting fragments in CD4^+^ T cells to interacting fragments found in erythroblasts or megakaryocytes. Both promoter fragments and, to a lesser extent, PIRs were enriched for histone modifications associated with transcriptionally active genes and regulatory elements (H3K27ac, H3K4me1, H3K4me3; Additional file 2: Figure S4) and changes in H3K27ac modifications at both promoter fragments and PIRs correlated with changes in gene expression upon activation. PIRs, but not promoter fragments, showed overlap with regions previously annotated as enhancers [16].

**Fig. 2:**
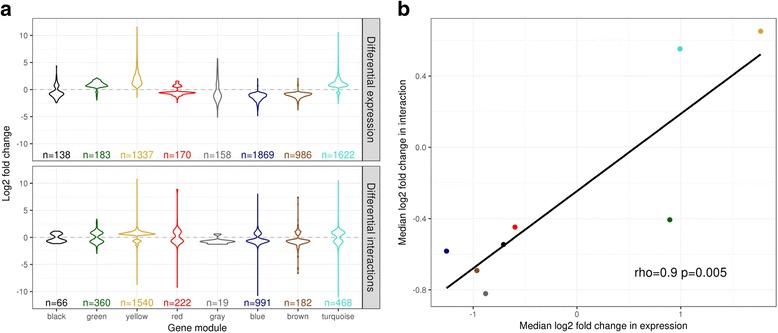
Change in PCHi-C interactions correlate with change in gene expression. **a** Distribution of significant (FDR < 0.01) fold changes induced by activation of CD4^+^ T cells in (*top*) gene expression and (*bottom*) differential PCHi-C interactions for differentially expressed genes in by module. **b** Median significant expression and interaction fold changes by module are correlated (Spearman rank correlation)

**Fig. 3:**
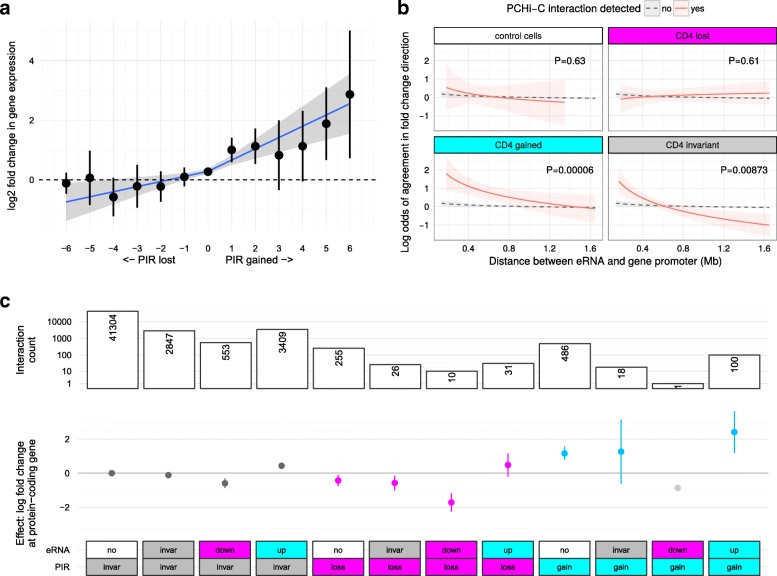
PCHi-C interactions and enhancer activity predict change in gene expression. **a** Change in gene expression at protein coding genes (log_2_ fold change, *y-axis*) correlates with the number of PIRs gained or lost upon activation (*x-axis*). **b** Fold change at transcribed sequence within the intergenic subset of regulatory regions (‘eRNAs’) was more likely to agree with the direction of protein-coding gene fold change when the two are linked by PCHi-C (*red*) in activated CD4^+^ T cells compared to pairs of eRNA and protein-coding genes formed without regard to PCHi-C derived interactions (background, *grey*, *p* < 10^−4^). Interactions were categorised as control (present only in megakaryocytes and erythroblasts, our control cells), invariant (‘invar’; present in non-activated and activated CD4^+^ T cells), ‘loss’ (present in non-activated but not activated CD4^+^ T cells and significantly downregulated at FDR < 0.01) or ‘gain’ (present in activated but not non-activated CD4^+^ T cells and significantly upregulated at FDR < 0.01). **c** Gain or loss of PIRs upon activation predicts change in gene expression, with the estimated effect more pronounced if accompanied by upregulation or downregulation at an eRNA. Points show estimated effect on gene expression of each interaction and lines the 95% confidence interval. PIRs categorised as in (**b**). eRNAs categorised as no (undetected), invariant (‘invar’, detected in non-activated and activated CD4^+^ T cells, differential expression FDR ≥ 0.01), up (upregulated; FDR < 0.01) or down (downregulated; FDR < 0.01). *Bar plot* (*top*) shows the number of interactions underlying each estimate. Note that eRNA = down, PIR = gain (*light gray*) has only one observation so no confidence interval can be formed and is shown for completeness only

We found that absolute levels of gene expression correlated with the number of PIRs (Additional file 2: Figure S5a; rho, 0.81), consistent with recent observations [15, 17]. We defined a subset of PCHi-C interactions that were specifically gained or lost upon activation (2334 and 1866, respectively, FDR < 0.01) and found that the direction of change (gain or loss) at these differential interactions agreed with the direction of differential expression (upregulated or downregulated) at the module level (Fig. 2). We further found that dynamic changes in gene expression upon activation correlated with changing numbers of PIRs. Notably, the pattern was asymmetrical, with a gained interaction associating with approximately twofold the change associated with a lost interaction (Fig. 3a). Given the > 6-h median half-life of messenger RNAs (mRNAs) expressed in T cells [18] (Additional file 2: Figure S5b), it is possible that the relatively smaller changes associated with lost interactions are due to the persistence of downregulated transcripts at the early stages of T cell activation.

As we sequenced total RNA without a poly(A) selection step, we were able to detect regulatory region RNAs (regRNAs), which are generally non-polyadenylated and serve as a mark for promoter and enhancer activity [19]. We defined 6147 ‘expressed’ regRNAs (see ‘Methods’) that mapped within regulatory regions defined by a 15 state ChromHMM [20] model (Additional file 2: Figure S6) and validated them by comparison to an existing cap analysis of gene expression (CAGE) dataset [21] which has been successfully used to catalogue active enhancers [22]. We found 2888/3897 (74%) regRNAs expressed in non-activated cells overlap CAGE defined enhancers. This suggests that the combination of chromatin state annotation and total RNA-seq data presents an alternative approach to capture active enhancers.

Almost half (48%) of expressed regRNAs showed differential expression after activation (2254/681 upregulated/downregulated; FDR < 0.01). To determine whether activity at these regRNAs could be related to that at PCHi-C linked genes, we focused attention on a subset of 640 intergenic regRNAs, which correspond to a definition of eRNAs [23]. Of these, 404 (63%) overlapped PIRs detected in CD4^+^ T cells and we found significant agreement in the direction of fold changes at eRNAs and their PCHi-C linked protein-coding genes in activated CD4^+^ T cells (*p* < 0.0001, Fig. 3b). We also observed a synergy between regRNA expression and the estimated effect of a PIR on expression with a gain or loss of a PIR overlapping a differentially regulated regRNA having the largest estimated effect on gene expression (Fig. 3c), supporting a sequential model of gene activation [24]. While regRNA function is still unknown [23], our results demonstrate the detection, by PCHi-C, of condition-specific connectivity between promoters and enhancers involved coordinating gene regulation.

### PCHi-C-facilitated mapping of candidate disease-causal genes

**Fig. 4:**
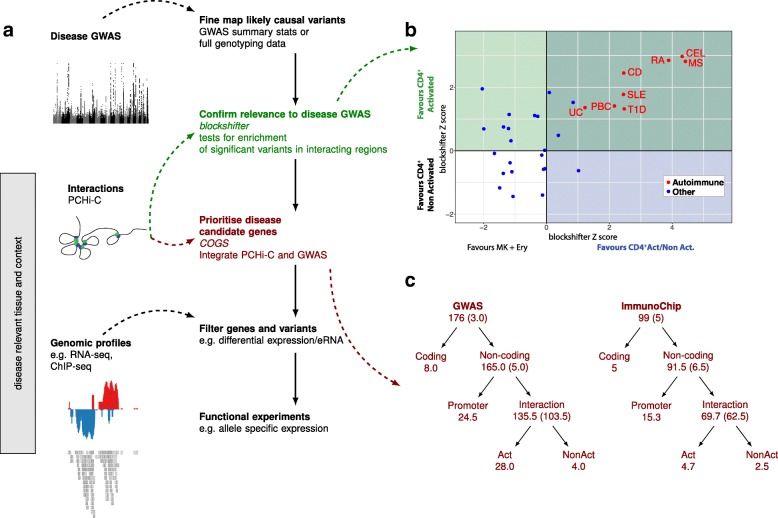
An experimental framework for identifying disease-causal genes. **a** Before prioritising genes, enrichment of GWAS signals in PCHi-C interacting regions should be tested to confirm the tissue and context are relevant to disease. Then, probabilistic fine-mapping of causal variants from the GWAS data can be integrated with the interaction data to prioritise candidate disease-causal genes, a list which can be iteratively filtered using genomic datasets to focus on (differentially) expressed genes and variants which overlap regions of open or active chromatin. **b** Autoimmune disease GWAS signals are enriched in PIRs in CD4^+^ T cells generally compared to control cells (blockshifter Z score, *x-axis*) and in PIRs in activated compared to non-activated CD4+ T cells (blockshifter Z score, *y-axis*). Text labels correspond to datasets described in Additional file 6: Table S5. **c** Genes were prioritised with a COGS score > 0.5 across five autoimmune diseases using genome-wide (GWAS) or targeted genotyping array (ImmunoChip) data. The numbers at each node give the number of genes prioritised at that level. Where there is evidence to split into one of two non-overlapping hypotheses (log_10_ ratio of gene scores > 3), the genes cascade down the tree. Act and NonAct correspond to gene scores derived using PCHi-C data only in activated or non-activated cells, respectively. Where the evidence does not confidently predict which of the two possibilities is more likely, genes are ‘stuck’ at the parent node (number given in brackets). When the same gene was prioritised for multiple diseases, we assigned fractional counts to each node, defined as the proportion of the n diseases for which the gene was prioritised at that node. Because of duplicate results between GWAS and ImmunoChip datasets, the total number of prioritised genes is 252 (see Table 1)

We defined an experimental framework to integrate PCHi-C interactions with GWAS data to map candidate disease-causal genes for autoimmune diseases (Fig. 4). First, to confirm that PCHi-C interactions inform interpretation of autoimmune disease GWAS, we tested whether PIRs were enriched for autoimmune disease GWAS signals by testing for different distributions of GWAS *p* values in PIRs of activated or non-activated CD4^+^ T cells compared to non-lymphoid cells (megakaryocytes and erythroblasts) and then in PIRs of activated compared to non-activated CD4^+^ T cells. To perform the test, we used *blockshifter* [17] which accounts for correlation between (1) neighbouring variants in the GWAS data and (2) neighbouring *Hind*III fragments in the interacting data by rotating one dataset with respect to the other, as previously proposed [25]. This method appropriately controls type 1 error rates, in contrast to methods based on counting associated SNP/PIRs which ignore correlation, such as a Fisher’s exact test (Additional file 2: Figure S7). We found autoimmune GWAS signals were enriched in CD4^+^ T cell PIRs compared to non-autoimmune GWAS signals (Wilcoxon *p* = 2.5 × 10^−7^) and preferentially so in activated vs. non-activated cells (Wilcoxon *p* = 4.8 × 10^−5^; Fig. 4).

Next, we fine-mapped causal variants for five autoimmune diseases using genetic data from a dense targeted genotype array, the ImmunoChip (ATD, CEL, RA, T1D) and summary data from GWAS data imputed to 1000 Genomes Project (RA, SLE; Additional file 6: Table S5). For the ImmunoChip datasets, with full genotype data, we also imputed to 1000 Genomes and used a Bayesian fine-mapping approach [26], which avoids the need for stepwise regression or assumptions of single causal variants and which provides a measure of posterior belief that any given variant is causal by aggregating posterior support over models containing that variant. Variant-level results are given in Additional files 7 and 8: Tables S6a and S6b, and show that of 73 regions with genetic association signals to at least one disease (minimum *p* < 5 × 10^−8^; 106 disease associations), nine regions have strong evidence that they contain more than one causal variant (posterior probability > 0.5), among them the well-studied region on chromosome 10 containing the candidate gene *IL2RA* [26]. For the GWAS summary data, we make the simplifying assumption that there exists a single causal variant in any LD-defined genetic region and again generate posterior probabilities that each variant is causal [27]. To integrate these variant level data with PCHi-C interactions and prioritise protein-coding genes as candidate causal genes for each autoimmune disease, for each gene, we aggregated posterior support over all models containing variants in PIRs for the gene promoter, within the promoter fragment or within its immediate neighbour fragments. Neighbouring fragments are included because of limitations in the ability of PCHi-C to detect very proximal interactions (within a region consisting of the promoter baited fragment and one *Hind*III fragment either side). The majority of gene scores were close to 0 (99% of genes have a score < 0.05) and we chose to use a threshold of 0.5 to call genes prioritised for further investigation. Having both ImmunoChip and summary GWAS data for RA allowed us to compare the two methods. Overlap was encouraging: of 24 genes prioritised for RA from ImmunoChip, 20 had a gene score > 0.5 in the GWAS prioritisation, and a further three had gene scores > 0.2. The remaining gene, *MDN1*, corresponded to a region which has a stronger association signal in the RA-ImmunoChip than RA-GWAS dataset, which may reflect the greater power of direct genotyping vs. imputation, given that the RA-ImmunoChip signal is echoed in ATD and T1D (Additional file 2: Figure S8). We prioritised a total of 245 unique protein-coding genes, 108 of which related to activation sensitive interactions (Additional files 9 and 10: Tables S7a and 7b, Fig. 4). Of 118 prioritised genes which could be related through interactions to a known susceptibility region, 63 (48%) lay outside that disease susceptibility region. The median distance from peak signal to prioritised gene was 153 kb. Note that prioritisation can be one (variant)-to-many (genes) because a single PIR can interact with more than one promoter and promoter fragments can contain more than one gene promoter. Note also that the score reflects both PCHi-C interactions and the strength and shape of association signals (Additional file 2: Figure S9), therefore a subset of prioritised genes relate to an aggregation over sub-genomewide significant GWAS signals. This is therefore a ‘long’ list of prioritised genes which requires further filtering (Table 1). A total of 179 (of 245) prioritised genes were expressed in at least one activation state; we highlight specifically the subset of 118 expressed genes which can be related to a genome-wide significant GWAS signal through proximity of a genome-wide significant SNP (*p* < 5 × 10^−8^) to a PIR. Of these, 82 were differentially expressed, 48 related to activation-sensitive interactions and 63 showed overlap of GWAS fine-mapped variants with an expressed eRNA (Additional file 9: Table S7a).

**Table 1:** Number of genes prioritised for autoimmune disease susceptibility under successive filters

Group	Description	Number of genes
1	Total	245
2	… Expressed	179
3	… … Proximal GWAS significant SNP (*p* < 5 × 10^−8^)	118
4	… … … Prioritised gene differentially expressed upon activation	82
5	… … … Prioritisation relates to activation sensitive interactions	48
6	… … … GWAS signal overlaps expressed regRNA in at least one state	63

Note that group 2 is a subset of group 1, group 3 is a subset of group 2, and groups 4, 5 and 6 are all subsets of group 3 but not necessarily of each other

Taken together, our results reflect the complexity underlying gene regulation and the context-driven effects that common autoimmune disease-associated variants may have on candidate genes. Our findings are consistent with, and extend, previous observations [7, 8] and we highlight six examples which exemplify both activation-specific and activation-invariant interactions.

### Exemplar regions

Here we highlight specific examples of prioritised genes with plausible autoimmune disease candidacy which illustrate three characteristics we found frequently, namely: (1) the identification of candidate genes some distance from association signals; (2) the tendency for multiple gene promoters to be identified as interacting with the same sets of disease-associated variants; and (3) genes prioritised in only one state of activation.

**Fig. 5:**
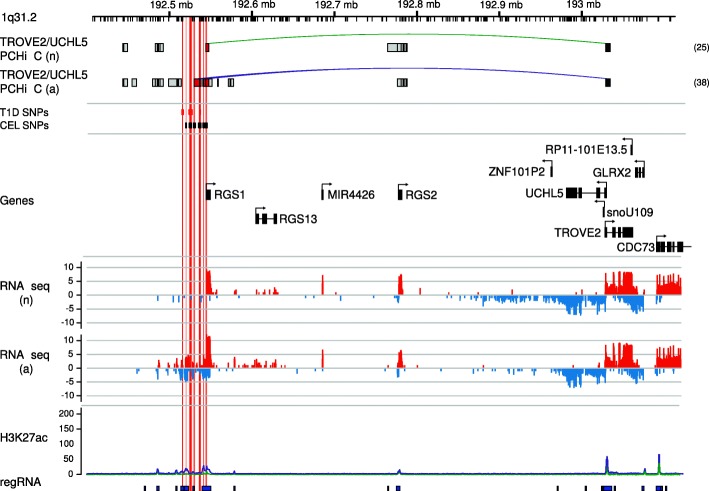
*TROVE2* and *UCLH5* on chromosome 1 are prioritised for CEL. The *ruler* shows chromosome location, with *Hind*III sites marked by ticks. The *top tracks* show PIRs for prioritised genes in non-activated (n) and activated (a) CD4^+^ T cells. *Green* and *purple lines* are used to highlight those PIRs containing credible SNPs from our fine-mapping. The total number of interacting fragments per PCHi-C bait is indicated in parentheses for each gene in each activation state. *Dark grey boxes* indicate promoter fragments; *light grey boxes*, PIRs containing no disease associated variants; and *red boxes*, PIRs overlapping fine-mapped disease-associated variants. The position of the fine-mapped variant area is indicated by *red boxes* and *vertical red lines*. Gene positions and orientation (Ensembl v75) are shown above log2 read counts for RNA-seq forward (*red*) and reverse (*blue*) strands. H3K27ac background-adjusted read count is shown in non-activated (*green line*) and activated (*purple line*) and boxes on the regRNA track show regions considered through ChromHMM to have regulatory marks

As an example of the first, CEL has been associated with a region on chromosome 1q31.2, for which *RGS1* has been named as a causal candidate due to proximity of associated variants to its promoter [28]. Sub-genome-wide significant signals for T1D (min. *p* = 1.5 × 10^−6^) across the same SNPs which are associated with CEL have been interpreted as a co-localising T1D signal in the region [29]. *RGS1* has recently been shown to have a role in the function of T follicular helper cells in mice [30], the frequencies of which and their associated IL-21 production have been shown to be increased in T1D patients [31]. However, our analysis also prioritises, in activated T cells, the strong functional candidate genes *TROVE2* and *UCHL5*, over half a megabase distant and with three intervening genes not prioritised (Fig. 5). *UCHL5* encodes ubiquitin carboxyl-terminal hydrolase-L5 a deubiquitinating enzyme that stabilises several Smad proteins and TGFBR1, key components of the TGF-beta1 signalling pathway [32, 33]. *TROVE2* is significantly upregulated upon activation (FDR = 0.005) and encodes Ro60, an RNA binding protein that indirectly regulates type-I IFN-responses by controlling endogenous Alu RNA levels [34]. A global anti-inflammatory effect for *TROVE2* expression would fit with its effects on gut (CEL) and pancreatic islets (T1D).

A similar situation is seen in the chromosome 1q32.1 region associated with T1D in which *IL10* has been named as a causal candidate gene [35]. Together with *IL10*, prioritised through proximity of credible SNPs to the *IL10* promoter, we prioritised other *IL10* gene family members *IL19*, *IL20* and *IL24* as well as two members of a conserved three-gene immunoglobulin-receptor cluster (*FCMR* and *PIGR*, Additional file 2: Figure S10). While *IL19*, *IL20* and *PIGR* were not expressed in CD4^+^ T cells, *FCMR* was downregulated and *IL24* and *IL10* were upregulated following CD4^+^ T cell activation. IL-10 is recognised as an important anti-inflammatory cytokine in health and disease [36] and candidate genes *FCMR* and *IL24* are components of a recently identified proinflammatory module in Th17 cells [37].

This region also exemplifies characteristic 2: a tendency for multiple gene promoters to be identified as interacting with the same sets of disease-associated variants. Parallel results have demonstrated co-regulation of multiple PCHi-C interacting genes by a single variant [37], suggesting that disease-related variants may act on multiple genes simultaneously, consistent with the finding that regulatory elements can interact with multiple promoters [38–40]. In this region, clusters of multiple adjacent PIRs were be detected for the same promoter fragments. It remains to be further validated whether all PIRs detected within such clusters correspond to ‘causal’ interactions or whether some such PIRs are ‘bystanders’ of strong interaction signals occurring in their vicinity. The use of PCHi-C nonetheless adds considerable resolution compared to simply considering topologically associating domains (TADs), which have a median length of 415 kb in naive CD4^+^ T cells [17] compared to a median of 37.5 kb total PIR length per gene in non-activated CD4^+^ T cells (Additional file 2: Figure S11).

Multiple neighbouring genes were also prioritised on chromosome 16q24.1: *EMC8*, *COX4I1* and *IRF8*, the last only in activated T cells, for two diseases: RA and SLE (Additional file 2: Figure S12). Candidate causal variants for SLE and RA fine-mapped to distinct PIRs, yet all these PIRs interact with the same gene promoters, suggesting that interactions, possibly specific to different CD4^+^ T cell subsets, may allow us to unite discordant GWAS signals for related diseases [6, 41, 42]. *EMC8* and *COX4I1* RNA expression was relatively unchanged by activation, whereas *IRF8* expression was upregulated 97-fold, coincident with the induction of 16 intergenic *IRF8* PIRs, four of which overlap autoimmune disease fine-mapped variants. Although the dominant effect of *IRF8* is to control the maturation and function of the mononuclear phagocytic system [43], a T cell-intrinsic function regulating CD4^+^ Th17 differentiation has been proposed [44]. Our data further link the control of Th17 responses to susceptibility to autoimmune disease including RA and SLE [45].

Another notable example, *AHR*, was one of the 38 genes we prioritised in only one state of activation (characteristic 3, Fig. 4): for RA, *AHR* was prioritised specifically in activated T cells rather than non-activated T cells (Additional file 2: Figure S13). *AHR* is a high affinity receptor for toxins in cigarette smoke that has been linked to RA previously through differential expression in synovial fluid of patients, though not through GWAS [46]. Our analysis prioritises *AHR* for RA due to a sub-genome-wide significant signal (rs71540792, *p* = 2.9 × 10^−7^) and invites attempts to validate the genetic association in additional RA patients.

### Interaction-mediated regulation of *IL2RA* expression

**Fig. 6:**
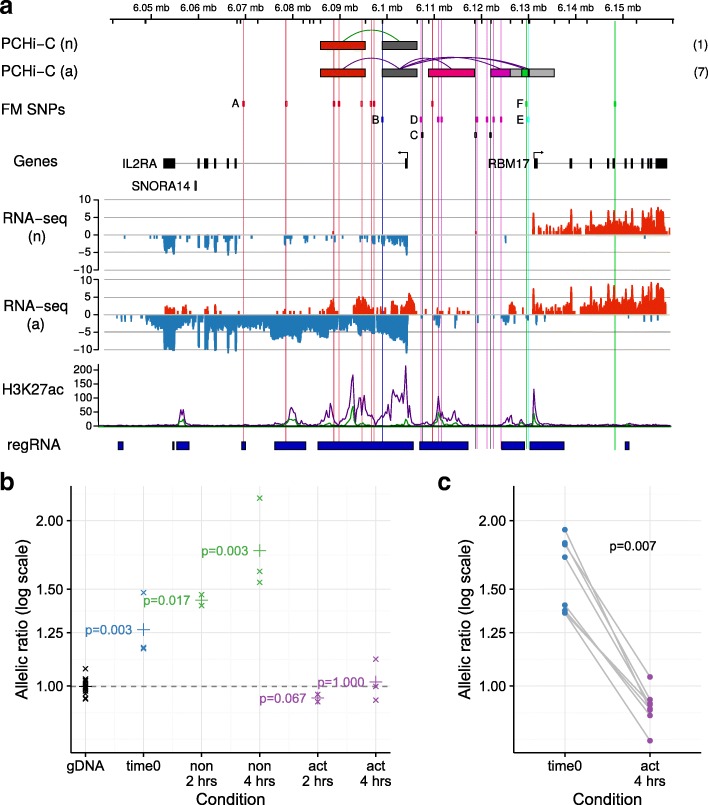
PCHi-C interactions link the *IL2RA* promoter to autoimmune disease-associated genetic variation, which leads to expression differences in *IL2RA* mRNA. **a** The *ruler* shows chromosome location, with *Hind*III sites marked by ticks. The *top tracks* show PIRs for prioritised genes in non-activated (n) and activated (a) CD4^+^ T cells. *Green* and *purple lines* are used to highlight those PIRs containing credible SNPs for the autoimmune diseases T1D and MS fine mapped on chromosome 10p15. Six groups of SNPs (A–F) highlighted in Wallace et al. [26] are shown, although note that group B was found unlikely to be causal. The total number of interacting fragments per PCHi-C bait is indicated in parentheses for each gene in each activation state. *Dark grey boxes* indicate promoter fragments; *light grey boxes*, PIRs containing no disease-associated variants; and *coloured boxes*, PIRs overlapping fine-mapped disease-associated variants. PCHi-C interactions link a region overlapping group A in non-activated and activated CD4+ T cells to the *IL2RA* promoter (*dark grey box*) and regions overlapping groups D and F in activated CD4+ T cells only. RNA-seq reads (log_2_ scale, *red* = forward strand, *blue* = reverse strand) highlight the upregulation of IL2RA expression upon activation and concomitant increases in H3K27ac (non-activated, n, *green line*; activated, a, *purple line*) in the regions linked to the *IL2RA* promoter. *Red vertical lines* mark the positions of the group A SNPs. Numbers in parentheses show the total number of IL2RA PIRs detected in each state. Here we show those PIRs proximal to the *IL2RA* promoter. Comprehensive interaction data can be viewed at https://www.chicp.org. **b** Allelic imbalance in mRNA expression in total CD4+ T cells from individuals heterozygous for group A SNPs using rs12722495 as a reporter SNP in non-activated (non) and activated (act) CD4^+^ T cells cultured for 2 or 4 h, compared to genomic DNA (gDNA, expected ratio = 1). Allelic ratio is defined as the ratio of counts of T to C alleles. ‘x’ = geometric mean of the allelic ratio over 2–3 replicates within each of 4–5 individuals; *p* values from a Wilcoxon rank sum test comparing complementary DNA (cDNA) to gDNA are shown. ‘+’ shows the geometric mean allelic ratio over all individuals. **c** Allelic imbalance in mRNA expression in memory CD4^+^ T cells differs between ex vivo (time 0) and 4-h activated samples from eight individuals heterozygous for group A SNPs using rs12722495 as a reporter SNP. *p* value from a paired Wilcoxon signed rank test is shown

Given our prior interest in the potential for autoimmune-disease associated genetic variants to regulate *IL2RA* expression [42], we were interested to note PCHi-C interactions between some of these variants and the *IL2RA* promoter. We attempted to confirm the predicted functional effects on *IL2RA* expression experimentally. *IL2RA* encodes CD25, a component of the key trimeric cytokine receptor that is essential for high-affinity binding of IL-2, regulatory T cell survival and T effector cell differentiation and function [47]. Multiple variants in and near *IL2RA* have been associated with a number of autoimmune diseases [35, 48–50]. We have previously fine-mapped genetic causal variants for T1D and multiple sclerosis (MS) in the *IL2RA* region [26], identifying five groups of SNPs in intron 1 and upstream of *IL2RA*, each of which is likely to contain a single disease-causal variant. Out of the group of eight SNPs previously denoted ‘A’ [26], three (rs12722508, rs7909519 and rs61839660) are located in an area of active chromatin in intron 1, within a PIR that interacts with the *IL2RA* promoter in both activated and non-activated CD4^+^ T cells (Fig. 6a). These three SNPs are also in LD with rs12722495 (r^2^ > 0.86) that has previously been associated with differential surface expression of CD25 on memory T cells [42] and differential responses to IL-2 in activated Tregs and memory T cells [51]. We measured the relative expression of *IL2RA* mRNA in five individuals heterozygous across all group A SNPs and homozygous across most other associated SNP groups, in a 4-h activation time-course of CD4^+^ T cells. Allelic imbalance was observed consistently for two reporter SNPs in intron 1 and in the 3’ UTR in non-activated CD4^+^ T cells in each individual (Fig. 6b; Additional file 2: Figure S14a), validating a functional effect of the PCHi-C-derived interaction between this PIR and the *IL2RA* promoter in non-activated CD4^+^ T cells. While the allelic imbalance was maintained in non-activated cells cultured for 2–4 h, the imbalance was lost in cells activated under our in vitro conditions. Since increased CD25 expression with rare alleles at group A SNPs has previously been observed on memory CD4^+^ T cells but not the naive or Treg subsets that are also present in the total CD4^+^ T cell population [42], we purified memory cells from eight group A heterozygous individuals and confirmed activation-induced loss of allelic imbalance of IL2RA mRNA expression in this more homogeneous population (Fig. 6c, Additional file 2: Figure S14b; Wilcoxon *p* = 0.007). *IL2RA* is one of the most strongly upregulated genes upon CD4^+^ T cell activation, showing a 65-fold change in expression in our RNA-seq data. Concordant with the genome-wide pattern (Fig. 3), the *IL2RA* promoter fragment gains PIRs that accumulate H3K27ac modifications upon activation and these, as well as potentially other changes marked by an increase in H3K27ac modification at rs61839660 and across the group A SNPs in intron 1, could account for the loss of allelic imbalance. These results emphasise the importance of steady-state CD25 levels on CD4^+^ T cells for the disease association mediated by the group A SNPs, levels which will make the different subsets of CD4^+^ T cells more or less sensitive to the differentiation and activation events caused by IL-2 exposure in vivo [52].

## Discussion

Our results illustrate the changes in chromosome conformation detected by PCHi-C in a single cell type in response to a single activation condition. That the PCHi-C technique can indeed link enhancers to their target genes is supported by our evidence that the direction of fold changes at eRNAs is connected to those at their PCHi-C linked protein-coding genes. Our results also provide support for the candidacy of certain genes and sequences in GWAS regions as causal for disease. Recent attempts to link GWAS signals to variation in gene expression in primary human cells have sometimes found only limited overlap [53–55]. One explanation may be that these experiments miss effects in specific cell subsets or states, especially given the transcriptional diversity between the many subsets of memory CD4^+^ T cells [56]. We highlight the complex nature of disease association at the *IL2RA* region where additional PIRs for *IL2RA* gained upon activation overlap other fine-mapped disease-causal variants (Fig. 6a), suggesting that other allelically imbalanced states may exist in activated cells, which may also correspond to altered disease risk. For example, the PIR containing rs61839660, a group A SNP, also contains an activation expression quantitative trait locus (eQTL) for *IL2RA* expression in CD4^+^ T effectors [57] marked by rs12251836, which is unlinked to the group A variants and was not associated with T1D [57]. Furthermore, rs61839660 itself has recently been reported as a QTL for methylation of the *IL2RA* promoter as well as an eQTL for *IL2RA* expression in whole blood [55, 58]. The differences between CD25 expression in different T cell subsets [59, 60] and the rapid activation-induced changes in gene and regulatory expression, chromatin marks and chromosome interactions we observe, imply that a large diversity of cell types and states will need to be assayed to fully understand the identity and effects of autoimmune disease-causal variants.

Our approach, like others such as eQTL analyses and integration of GWAS variants with chromatin state information, offers a view of disease through the prism of purified cell subsets in specific states of activation. However, a more complete range of cell types and activation states will be needed for the comprehensive understanding of complex diseases for which multiple cell types are aetiologically involved. It will be challenging to assay this greater diversity of cell types and states in the large numbers of individuals needed for traditional eQTL studies, particularly for cell-type or condition-specific eQTLs that have been shown to generally have weaker effects [61, 62]. Allele-specific expression (ASE) is a more powerful design to quantify the effects of genetic variation on gene expression with modest sample sizes [63] and the targeted ASE approach that we adopt enables testing of individual variants or haplotypes at which donors are selected to be heterozygous, while controlling for other potentially related variants at which donors are selected to be homozygous.

## Conclusions

Here we have presented an approach for connecting disease-associated variants derived from GWAS with putative target genes based on promoter interactome maps obtained with PCHi-C. By using statistical fine-mapping of GWAS data, integrated with PCHi-C, to highlight both likely disease-causal variants and their potential target genes, we enable the design of targeted ASE analyses for functional confirmation of individual effects. This systematic experimental framework offers an alternative approach to candidate causal gene identification for variants with cell state-specific functional effects, with achievable sample sizes.

## Methods

### CD4^+^ T cell purification and activation, preparation for genomics assays

Blood samples were obtained from healthy donors selected from the Cambridge BioResource. Donors were excluded if they were diagnosed with autoimmune disease or cancer, were receiving immunosuppressants, cytotoxic agents or intravenous immunoglobulin or had been vaccinated or received antibiotics in the 2–4 weeks preceding the blood donation. CD4^+^ T cells were isolated from whole blood using RosetteSep (STEMCELL technologies, Canada) according to the manufacturer’s instructions. Purified CD4^+^ T cells (average, 96.5% pure; range, 92.9–98.7%) were washed in X-VIVO 15 supplemented with 1% AB serum (Lonza, Switzerland) and penicillin/streptomycin (Invitrogen, UK) and plated in 96-well CELLSTAR U-bottomed plates (Greiner Bio-One, Austria) at a concentration of 2.5 × 10^5^ cells/well. Cells were left untreated or stimulated with Dynabeads human T activator CD3/CD28 beads (Invitrogen, UK) at a ratio of 1 bead : 3 cells for 4 h at 37 °C and 5% CO_2_. Cells were harvested, centrifuged, supernatant removed and either: (1) resuspended in RLT buffer (RNeasy micro kit, Qiagen, Germany) for RNA-seq (0.75-1 × 10^6^ CD4^+^ T cells/pool and activation state); or (2) fixed in formaldehyde for capture Hi-C (44–101 × 10^6^ CD4^+^ T cells/pool and activation state) or ChIP-seq (16–26 × 10^6^ CD4^+^ T cells/pool and activation state) as detailed in [17].

ChIP-seq (H3K27ac, H3K4me1, H3K4me3, H3K27me3, H3K9me3, H3K36me3) was carried out according to BLUEPRINT protocols [17]. Formaldehyde fixed cells were lysed, sheared and DNA sonicated using a Bioruptor Pico (Diagenode). Sonicated DNA was pre-cleared (Dynabeads Protein A, Thermo Fisher) and ChIP performed using BLUEPRINT validated antibodies and the IP-Star automated platform (Diagenode). Libraries were prepared and indexed using the iDeal library preparation kit (Diagenode) and sequenced (Illumina HiSeq, paired-end).

For PCHi-C [17], DNA was digested overnight with *Hind*III, end-labelled with biotin-14-dATP and ligated in preserved nuclei. De-crosslinked DNA was sheared to an average size of 400 bp, end-repaired and adenine-tailed. Following size selection (250–550 bp fragments), biotinylated ligation fragments were immobilised, ligated to paired-end adaptors and libraries amplified (7–8 polymerase chain reaction [PCR] amplification rounds). Biotinylated 120-mer RNA baits targeting both ends of *Hind*III restriction fragments that overlap Ensembl-annotated promoters within the Ensembl categories of *protein-coding*, *non-coding*, *antisense*, *snRNA*, *miRNA* and *snoRNA* were used to capture targets [64]. After enrichment, the library was further amplified (four PCR cycles) and sequenced on the Illumina HiSeq 2500 platform. Each PCHi-C library was sequenced over three lanes generating 50-bp paired-end reads.

### PCHi-C interaction calls

Raw sequencing reads were processed using the HiCUP pipeline [65] and interaction confidence scores were computed using the CHiCAGO pipeline [14] as previously described [17]. We considered the set of interactions with high confidence scores (>5) in this paper.

Raw PCHi-C read counts from three replicates and two conditions were transformed into a matrix and a trimmed mean of M-values normalisation was applied to account for library size differences. Subsequently, a voom normalisation was applied to log-transformed counts in order to estimate precision weights per contact and differential interaction estimates were obtained after fitting a linear model on a paired design, using the limma Bioconductor R package [66].

### Microarray measurement of gene expression

We recruited 20 healthy volunteers from the Cambridge BioResource. Total CD4^+^ T cells were isolated from whole blood within 2 h of venepuncture by RosetteSep (StemCell Technologies). To assess the transcriptional variation in response to TCR stimulation, 10^6^ CD4^+^ T cells were cultured in U-bottom 96-well plates in the presence or absence of human T activator CD3/CD28 beads at a ratio of 1 bead : 3 cells. Cells were harvested at 2, 4, 6 or 21 h post stimulation or after 0, 6 or 21 h in the absence of stimulation. Three samples from the 6-h unstimulated time point were omitted from the study due to insufficient cell numbers and a further four samples were dropped after quality control, resulting in a total of 133 samples that were included in the final analysis. RNA was isolated using the RNAeasy kit (Qiagen) according to the manufacturer’s instructions.

cDNA libraries were synthesised from 200 ng total RNA using a whole-transcript expression kit (Ambion) according to the manufacturer’s instructions and hybridised to Human Gene 1.1 ST arrays (Affymetrix). Microarray data were normalised using a variance stabilising transformation [67] and differential expression was analysed in a paired design using limma [66]. Genes were clustered into modules using WGCNA [68].

### ChIP-sequencing and regulatory annotation

ChIP-seq reads for all histone modification assays and control experiments were mapped to the reference genome using BWA-MEM [69], a Burrows-Wheeler genome aligner. Samtools [70] was employed to filter secondary and low-quality alignments (we retained all read pair alignments with PHRED score > 40 that matched all bits in SAM octal flag 3 and did not match any bits in SAM octal flag 3840). The remaining alignments were sorted, indexed and a whole-genome pileup was produced for each histone modification, sample and condition triple.

We used ChromHMM [20], a multivariate hidden Markov model, to perform a whole-genome segmentation of chromatin states for each activation condition. First, we binarised read pileups for each chromatin mark pileup using the corresponding control experiment as a background model. Second, we estimated the parameters of a 15-state hidden Markov model (a larger state model resulted in redundant states) using chromosome 1 data from both conditions. Parameter learning was re-run five times using different random seeds to assess convergence. Third, a whole-genome segmentation was produced for each condition by running the obtained model on the remaining chromosomes. Each state from the obtained model was manually annotated and states indicating the presence of promoter or enhancer chromatin tags were selected (E4–E11, Additional file 2: Figure S6). Overlapping promoter or enhancer regions in non-activated and activated genome segmentations were merged to create a CD4^+^ T cell regulatory annotation. Thus, we defined 53,534 regulatory regions (Additional files 11, 12 and 13: Tables S8a–S8c).

### RNA sequencing

Total RNA was isolated using the RNeasy kit (Qiagen) and the concentrations and integrity were quantified using Bioanalyzer (Agilent); all samples reached RINs > 9.8. Two pools of RNA were generated from three and four donors and for each experimental condition. cDNA libraries were prepared from 1 ug total RNA using the stranded NEBNext Ultra Directional RNA kit (New England Biolabs) and sequenced on HiSeq (Illumina) at an average coverage of 38 million paired-end reads/pool. RNA-seq reads were trimmed to remove traces of library adapters by matching each read with a library of contaminants using Cutadapt [71], a semi-global alignment algorithm. Owing to our interest in detecting functional enhancers, which constitute transcription units on their own right, we mapped reads to the human genome using STAR [72], a splicing-aware aligner. This frees us from relying on a transcriptome annotation which would require exact boundaries and strand information for all features of interest, something not available in case of promoters and enhancers.

After alignment, we employed Samtools [70] to discard reads with an unmapped pair, secondary alignments and low-quality alignments. The resulting read dataset, with an average of 33 million paired-end reads/sample, was sorted and indexed. We used FastQC (v0.11.3, http://www.bioinformatics.babraham.ac.uk/projects/fastqc/) to ensure all samples had regular GC content (sum of deviations from normal includes less than 15% of reads), base content per position (difference A vs. T and G vs. C less than 10% at all positions) and kmer counts (no imbalance of kmers with *p* < 0.01) as defined by the tool. We augmented Ensembl 75 gene annotations with regulatory region definitions obtained from our ChIP-seq analysis described above and defined them as present in both genome strands due to their bi-directional transcription potential. For each RNA-seq sample, we quantified expression of genomic and regulatory features in a two-step strand-aware process using HTSeq. [73] For each gene we counted the number of reads that fell exactly within its exonic regions and did not map to other genomic elements. For each regulatory feature, we counted the number of reads that fell exactly within its defined boundaries and did not map to other genomic or regulatory elements.

By construction, this quantification scheme counts each read at most once towards at most one feature. Furthermore, strand information is essential to be able to assign reads to features in regions with overlapping annotations. For example, distinguishing intronic eRNAs from pre-mRNA requires reads originating from regulatory activity in the opposite strand from the gene.

Feature counts were transformed into a matrix and a trimmed mean of M-values normalisation was applied to account for library size differences, plus a filter to discard features below an expression threshold of < 0.4 counts per million mapped reads in at least two samples, a rather low cutoff, to allow for regulatory RNAs to enter differential expression calculations. This threshold equates to approximately 15 reads, given our mapped library sizes of ~35 million paired-end reads. A voom normalisation was applied to log-transformed counts in order to estimate precision weights per gene and differential expression estimates were obtained after fitting a linear model on a paired design, using the limma Bioconductor R package [66]. There was a strong correlation (rho = 0.81) between microarray and RNA-seq fold change estimates at 4 h.

### Comparison of regRNAs to FANTOM CAGE data

We compared expressed regRNA regions detected in our non-activated CD4^+^ T cell samples vs. those found using CAGE-seq by the FANTOM5 Consortium. RNA-seq, using a regulatory reference obtained from chromatin states, yields 17,175 features expressed with at least 0.4 counts per million in both non-activated CD4^+^ T cell samples. Among those, 3897 correspond to regulatory regions. Unstimulated CD4^+^ samples from FANTOM5 (http://fantom.gsc.riken.jp/5/datafiles/latest/basic/human.primary_cell.hCAGE/, samples 10,853, 11,955 and 11998) contain 266,710 loci expressed (with at least one read) in all three samples.

We found 13,178 of our 17,175 expressed CD4^+^ T cell features overlap expressed loci in CAGE data (77%). Conversely, 243,596/266,710 CAGE loci overlap CD4^+^ T cell features (91%). Similarly, 2888/3897 expressed regRNAs overlap expressed loci in CAGE data (74%).

### Comparison of PCHi-C and ChIA-PET interactions

We downloaded supplementary Table 1 from http://www.nature.com/cr/journal/v22/n3/extref/cr201215x1.xlsx [15] and counted the overlaps of PCHi-C interactions from CD4^+^ T cells and comparitor cells (megakaryoctyes and erythroblasts) in distance bins. R code to replicate the analysis is at https://github.com/chr1swallace/cd4-pchic/blob/master/analyses/chepelev-comparison.R. Calling interactions requires correction for the expected higher density of random collisions at shorter distances [74] which are explicitly modelled by CHICAGO [14] used in this study but not in the ChIA-PET study [15]. As a result, we expected a higher false-positive rate from the ChIA-PET data at shorter distances.

### Regression of gene expression against PIR count and eRNA expression

We related measures of gene expression (absolute log2 counts or log2 fold change) to numbers of PIRs or numbers of PIRs overlapping specific features using linear regression. We used logistic regression to relate agreement between fold change direction at PCHi-C linked protein-coding genes and eRNAs. We used robust clustered variance estimates to account for the shared baits for some interactions across genes with the same prey. Enrichment of chromatin marks in interacting baits and prey were assessed by logistic regression modelling of a binary outcome variable (fragment overlapped specific chromatin peak) against a fragment width and a categorical explanatory variable (whether the *Hind*III fragment was a bait or prey and the cell state the interaction was identified in), using block bootstrapping of baited fragments (https://github.com/chr1swallace/genomic.autocorr) to account for spatial correlation between neighbouring fragments.

### GWAS summary statistics

We used a compendium of 31 GWAS datasets [17] (Additional file 6: Table S5). Briefly, we downloaded publicly available GWAS statistics for 31 traits. Where necessary we used the *liftOver* utility to convert these to GRCh37 coordinates. To remove spurious association signals, we removed variants with *p* < 5 × 10^−8^ for which there were no variants in LD (r^2^ > 0.6 using 1000 genomes EUR cohort as a reference genotype panel) or within 50 kb with *p* < 10^−5^. We masked the MHC region (GRCh37:chr6:25-35 Mb) from all downstream analysis due to its extended LD and known strong and complex associations with autoimmune diseases.

Comparison of GWAS data and PIRs requires dense genotyping coverage. For GWAS which did not include summary statistics imputed for non-genotyped SNPs, we used a poor man’s imputation (PMI) method [17] to impute. We imputed *p* values at ungenotyped variants from 1000 Genomes EUR phase 3 by replacing missing values with those of their nearest proxy variant with r^2^ > 0.6, if one existed. Variants that were included in the study but did not map to the reference genotype set were also discarded.

To calculate posterior probabilities that each SNP is causal under a single causal variant assumption, we divided the genome into linkage disequilibrium blocks of 1 cM based on data from the HapMap project (ftp://ftp.ncbi.nlm.nih.gov/hapmap/recombination/2011-01_phaseII_B37/). For each region excluding the MHC we used code modified from Giambartolomei et al. [75] to compute approximate Bayes factors for each variant using the Wakefield approximation [76]; thus, posterior probabilities that each variant was causal as previously proposed [77]. The method assumes a normal prior on the population log relative risk centred at 0 and we set the variance of this distribution to 0.04, equivalent to a 95% belief that the true relative risk is in the range of 0.66–1.5 at any causal variant. We set the prior probability that any variant is causal for disease to 10^−4^.

### Testing of the enrichment of GWAS summary statistics in PIRs using *blockshifter*

We used the *blockshifter* method [17] (https://github.com/ollyburren/CHIGP) to test for a difference between variant posterior probability distributions in *Hind*III fragments with interactions identified in test and control cell types using the mean posterior probability as a measure of central location. *Blockshifter* controls for correlation within the GWAS data due to LD and interaction restriction fragment block structure by employing a rotating label technique similar to that described in GoShifter [25] to generate an empirical distribution of the difference in means under the null hypothesis of equal means in the test and control set. Runs of one or more PIRs (separated by at most one *Hind*III fragment) are combined into ‘blocks’, that are labelled unmixed (either test or control PIRs) or mixed (block contains both test and control PIRs). Unmixed blocks are permuted in a standard fashion by reassigning either test or control labels randomly, taking into account the number of blocks in the observed sets. Mixed blocks are permuted by conceptually circularising each block and rotating the labels. A key parameter is the gap size—the number of non-interacting *Hind*III fragments allowed within a single block, with larger gaps allowing for more extended correlation.

We used simulation to characterise the type 1 error and power of *blockshifter* under different conditions and to select an optimal gap size. First, from the Javierre et al. dataset [17], we selected a test (Activated or Non Activated CD4^+^ T Cells) and control (Megakaryocyte or Erythroblast) set of PIRs with a CHiCAGO score > 5, as a reference set for *blockshifter* input.

Using the European 1000 genomes reference panel, we simulated GWAS summary statistics, under different scenarios of GWAS/PIR enrichment. We split chromosome 1 into 1 cM LD blocks and used reference genotypes to compute a covariance matrix for variants with minor allele frequency above 1%, Σ. GWAS Z scores can be simulated as multivariate normal with mean μ and variance Σ [78]. Each block may contain no causal variants (GWAS_null_, μ = 0) or one (GWAS_alt_). For GWAS_alt_ blocks, we pick a single causal variant, *i*, and calculate the expected non-centrality parameter (NCP) for a 1 degree of freedom chi-square test of association at this variant and its neighbours. This framework is natural because, under a single causal variant assumption, the NCP at any variant *j* can be expressed as the NCP at the causal variant multiplied by the r^2^ between variants *i* and *j* [79]. In each case, we set the NCP at the causal variant to 80 to ensure that each causal variant was genome-wide significant (*p* < 5 × 10^−8^). μ is defined as the square root of this constructed NCP vector.

For all scenarios, we randomly chose 50 GWAS_alt_ blocks leaving the remaining 219 GWAS_null_. Enrichment is determined by the preferential location of simulated causal variants within test PIRs. In all scenarios, each causal variant has a 50% chance of lying within a PIR, to mirror a real GWAS in which we expect only a proportion of causal variants to be regulatory in any given cell type. Under the enrichment-null scenario, used to confirm control of type 1 error rate, the remaining variants were assigned to PIRs without regard for whether they were identified in test or control tissues. To examine power, we considered two different scenarios with PIR-localised causal variants chosen to be located specifically in test PIRs with either 50% probability, scenario power (1) or 100%, scenario power (2). Note that a PIR from the test set may also be in the control set, thus, as with a real GWAS, not all causal variants will be informative for this test of enrichment.

For each scenario, we further considered variable levels of genotyping density, corresponding to full genotyping (everything in 1000 Genomes), HapMap imputation (the subset of SNPs also in Stahl et al. [80] dataset) or genotyping array (the subset of SNPs also on the Illumina 550 k array). Where genotyping density is less than full, we used our proposed PMI strategy to fill in Z scores for missing SNPs.

We ran *blockshifter*, with 1000 null permutations, for each scenario and PMI condition for 4000 simulated GWAS, with a *blockshifter* superblock gap size parameter (the number of contiguous non-PIR *Hind*III fragments allowed within one superblock) of between 1 and 20 and supplying numbers of cases and controls from the RA dataset [49].

For comparison, we also investigated the behaviour of a naive test for enrichment for the null scenario. We computed a 2 × 2 table variants according to test and control PIR overlap, and whether a variant’s posterior probability of causality exceeded an arbitrary threshold of 0.01, and Fisher’s exact test to test for enrichment.

### Enrichment of GWAS summary statistics in CD4^+^ and activated CD4^+^ PIRs

We compared the following sets using all GWAS summary statistics, with a superblock gap size of 5 (obtained from simulations above) and 10,000 permutations under the null:

- Total CD4^+^ Activated + Total CD4^+^ NonActivated (test) vs. endothelial precursors + megakaryocytes (control)
- Total CD4^+^ Activated (test) vs. Total CD4^+^ NonActivated (control)

### Variant posterior probabilities of inclusion, full genotype data (ImmunoChip)

We carried out formal imputation to 1000 Genomes Project EUR data using IMPUTE2 [81] and fine-mapped causal variants in each of the 179 regions where a minimum *p* < 0.0001 was observed using a stochastic search method which allows for multiple causal variants in a region (https://github.com/chr1swallace/GUESSFM) [26]. Despite the pre-selection of regions associating with autoimmune diseases on the ImmunoChip, we chose to again set the prior probability that any variant was causal to 10^−4^, to align our analysis with that applied to the GWAS summary data. The prior probability for individual models follows a binomial distribution, according to the number of causal variants represented, so that the prior for each of the (^*n*^_*k*_) *k*- SNP causal variant models was (10^−4^)^*k*^(1–10^−4^)^(*n-k*)^ where *n* is the number of SNPs considered in the region. The posterior probabilities for models that contained variants which overlapped PIRs for each gene were aggregated to compute PIR-level marginal posterior probabilities of inclusion.

### Variant posterior probabilities of inclusion, summary statistics

Where we have only summary statistics of GWAS data already imputed to 1000 Genomes, we divided the genome into linkage disequilibrium blocks of 0.1 cM based on data from the HapMap project (ftp://ftp.ncbi.nlm.nih.gov/hapmap/recombination/2011-01_phaseII_B37/). For each region, excluding the MHC, we use code modified from Giambartolomei et al. [75] to compute approximate Bayes factors for each variant using the Wakefield approximation [76]; thus, posterior probabilities that each variant was causal assuming at most one causal variant per region as previously proposed [77].

### Computation of gene prioritisation scores

We used the COGS method [17] (https://github.com/ollyburren/CHIGP) to prioritise genes for further analysis. We assign variants to the first of the following three categories it overlaps for each annotated gene, if any:

1. coding variant: the variant overlaps the location of a coding variant for the target gene;
2. promoter variant: the variant lies in a region baited for the target gene or adjacent restriction fragment;
3. PIR variant: the variant lies in a region overlapping any PIR interacting with the target gene.

We produced combined gene/category scores by aggregating, within LD blocks, over models with a variant in a given set of PIRs (interacting regions) or over *Hind*III fragments baited for the gene promoter and immediate neighbours (promoter regions) or over coding variants to generate marginal probabilities of inclusion (MPPI) for each hypothesised group. We combine these probabilities across LD blocks, *i*, using standard rules of probability to approximate the posterior probability that at least one LD block contains a causal variant:

$$ \mathrm{gene}\  \mathrm{score}=1-\prod_{i\in \mathrm{LD}\ \mathrm{blocks}}\left(1-\left[\mathrm{score}\ \mathrm{for}\ i\right]\right) $$

Thus, the score takes a value between 0 and 1, with 1 indicating the strongest support. We report all results with score > 0.01 in Additional file 9: Table S7b, but focus in this manuscript on the subset with scores > 0.5.

Because COGS aggregates over multiple signals, a gene may be prioritised because of many weak signals or few strong signals in interacting regions. To estimate the expected number of prioritised genes for a typical GWAS signal, we considered the subset of 76 input regions with genome-wide significant signals (*p* < 5 × 10^−8^) in ImmunoChip datasets. We prioritised at least one gene with a COGS score > 0.5 in 35 regions, with a median of three genes/region (interquartile range [IQR] = 1.5-4). Equivalent analysis of the genome-wide significant GWAS signals prioritised a median of two genes/region (IQR = 1–3). This suggests that this algorithm might be expected to prioritise at least one gene in about half the genome-wide significant regions input when run on a relevant cell type.

We developed a hierarchical heuristic method to ascertain for each target gene, which was the mostly likely component and cell state. First, for each gene we compute the gene score due to genic variants (components 1 + 2) and variants in PIRs (component 3) using all available tissue interactions for that gene. While components 1 and 2 are fixed for a given gene and trait, the contribution of variants overlapping PIRs varies depending on the tissue context being examined. We use the ratio of the genic score to PIRs score in a similar manner to a Bayes factor to decide whether causal variants contributing to the gene score are more likely to lie within the gene or within its associated PIRs. If a genic location is more likely (gene.score ratio > 3) we iterate and compare if the gene score due to coding variants (component 1) is more likely than for promoter variants (component 2). Similarly, if PIRs are more likely, we compare PIR gene scores for activated vs. non-activated cells. If at any stage no branch is substantially preferred over its competitor (ratio of gene scores < 3), we return the previous set as most likely, otherwise we continue until a single cell state/set is chosen. In this way, we can prioritise genes based on the overall score and label as to a likely mechanism for candidate causal variants.

### Allele-specific expression assays

Total CD4^+^ T cells were isolated from five healthy donors and activated as described above and were harvested after 0, 2 and 4 h in RLT Plus buffer. Selected donors were heterozygous for all eight group A SNPs and homozygous for group C and F SNPs. Two and three of the donors were homozygous for the group D and E SNP groups, respectively (Additional file 14: Table S9). Memory CD4^+^ T cells were sorted from cryopreserved PBMC from an additional eight healthy donors as viable, αβ TCR^+^, CD4^+^, CD45RA^−^, CD127^+^ and CD27^+^ cells using a FACSAria III cell sorter (BD Biosciences). Sorted cells were either activated for 4 h in culture as described above or resuspended directly in RLT plus buffer post-sort. Total RNA was extracted using Qiagen RNeasy Micro plus kit and cDNA was synthesised using Superscript III reverse transcriptase (Thermo Fisher) according to the manufacturer’s instructions. To perform allele-expression experiments we used a modified version of a previously described method for quantifying methylation in bisulphite sequence data [82]. A two-stage PCR was used, the first round primers were designed to flank the variant of interest using Primer3 (http://bioinfo.ut.ee/primer3-0.4.0/primer3/) and adaptor sequences were added to the primers (Sigma), shown as lowercase letters (rs61839660_ASE_F tgtaaaacgacggccagtGCACACACCTATCCTAGCCT, rs61839660_ASE_R caggaaacagctatgaccCCCACAGAATCACCCACTCT, product size 114 bp; rs12244380_ASE_F tgtaaaacgacggccagtTTCGTGGGAGTTGAGAGTGG, rs12244380_ASE_R caggaaacagctatgaccTTAAAAGAGTTCGCTGGGCC, product size 180 bp; rs12722495_ASE_F tgtaaaacgacggccagtGTGAGTTTCAATCCTAAGTGCGA, rs12722495_ASE_R caggaaacagctatgaccATTAAGCGGACTCTCTGGGG, product size 97 bp). The first-round PCR contains 10 μL of Qiagen multiplex PCR mastermix, 0.5 μL of 10 nmol forward primer, 0.5 μL of 10 nmol reverse primer, 4 μL of cDNA and made up to 20 μL with ultra-pure water. The PCR cycling conditions were 95 °C for 15 min hot start, followed by 30 cycles of the following steps: 95 °C for 30 s, 60 °C for 90 s and 72 °C for 60 s, finishing with a 72 °C for 10-min cycle. The first-round PCR product was cleaned using AmpureXP beads (Beckman Coulter) according to manufacturer’s instructions. To add Illumina sequence compatible ends to the individual first-round PCR amplicons, additional primers were designed to incorporate P1 and A sequences plus sample-specific index sequences in the A primer, through hybridisation to adapter sequence present on the first-round gene-specific primers. Index sequences are as published [82]. The second-round PCR contained 8 μL of Qiagen multiplex PCR mastermix, 2.0 μL of ultra-pure water, 0.35 μL of each forward and reverse index primer and 5.3 μL of Ampure XP-cleaned first-round PCR product. The PCR cycling conditions were 95 °C for 15 min hot start, followed by seven cycles of the following steps: 95 °C for 30 s, 56 °C for 90 s, 72 °C for 60 s, finishing with 72 °C for a 10-min cycle. All PCR products were pooled at equimolar concentrations based on quantification on the Shimadzu Multina. AmpureXP beads were used to remove unincorporated primers from the product pool. We used the Kapa Bioscience library quantification kit to accurately quantify the library according to the manufacturer’s instructions before sequencing on an Illumina MiSeq v3 reagents (2 × 300 bp reads).

### Statistical analysis of allele-specific expression data

Sequence data were processed using the Methpup package (https://github.com/ollyburren/Methpup) to extract counts of each allele at rs12722495 and rs12244380 (Additional file 15: Table S10). Individuals were part of a larger cohort genotyped on the ImmunoChip and were phased using snphap (https://github.com/chr1swallace/snphap) to confirm which allele at each SNP was carried on the same chromosome as A2 = rs12722495:C or A1 = rs12722495:T. Allelic imbalance was quantified as the ratio A2/A1 and was averaged across replicates within individuals using a geometric mean. Allelic ratios in cDNA and gDNA were compared using Wilcoxon rank sum tests. *p* values are shown in Fig. 6b and Additional file 2: Figure S14. Full details are in https://github.com/chr1swallace/cd4-pchic/blob/master/ASE/IL2RA-ASE.R.

## Additional files

Additional file 1: Table S1. Gene modules inferred from WGCNA analysis of microarray time-course. Expression fold changes and associated false discovery rates (adjusted *p* values) are from RNA-seq data at the 4-h timepoint. (GZ 278 kb)

Additional file 2: Figures S1–S14. **Figure S1.** Comparison of longer and shorter CD4+ T cell activation timecourses. **Figure S2.** Summary distributions of interacting fragments. **Figure S3.** Validation of PCHi-C by ChIA-PET. **Figure S4.** Chromatin state profiles of interacting fragments. **Figure S5.** Relationship of gene expression to PIR number and mRNA half-life. **Figure S6.** Definition and quantification of regulatory RNAs. **Figure S7.** blockshifter calibration. **Figure S8.** *MDN1* is prioritised for RA through ImmunoChip but not GWAS data. **Figure S9.** Gene prioritisation using COGS. **Figure S10.** Multiple genes on chromosome 1q32.1 (*IL10, IL19, IL20, IL24, FCAMR/PIGR*) are prioritised for T1D, CRO and UC. **Figure S11.** Histograms show the distribution of summed PIR length by gene in non-activated CD4+ T cells (top panel) and TAD length in naive CD4+ T cells. **Figure S12.** *IRF8* and *EMC8/COX4I1* on chromosome 16 are prioritised for RA and SLE. **Figure S13.** *AHR* on chromosome 7 is prioritised for RA in activated CD4+ T cells. **Figure S14.** Allelic imbalance in mRNA expression in individuals heterozygous for group A SNPs is confirmed with reporter SNP rs12244380 (*IL2RA* 3’ UTR). (PDF 4243 kb)

Additional file 3: Table S2. Results of differential expression analysis on RNA-seq data. Features are defined in the GTF file in Additional file 11: Table S8a. (GZ 835 kb)

Additional file 4: Table S3. Baited HindIII fragments used for capture of Hi-C libraries, annotated with Ensembl annotated genes. (GZ 572 kb)

Additional file 5: Table S4. PCHi-C interactions called with the CHiCAGO pipeline. Annotation for baited fragments is given in Additional file 4: Table S3. PIRs are called other ends (‘oe’). CHICAGO scores for activated (‘Total_CD4_Activated’) and non-activated (‘Total_CD4_NonActivated’) CD4+ T cells were considered called with confidence if above 5. We also conducted differential analysis, and the read counts input into that are given by the columns P1.non - P3.act, with the results summarised by their log fold change (logFC) and FDR. Bait-PIR pairs are shown only if the CHiCAGO score is ≥ 5 for at least one CD4+ T cell. (GZ 14529 kb)

Additional file 6: Table S5. Summary of GWAS data used. ‘type’ indicates whether the trait was quantitative (QUANT) or case/control (CC). For CC, ‘cases’ and ‘controls’ columns represent the number of individuals included in the study, while for QUANT, the number of individuals is given in the cases column. ‘Category’ indicates broader classes of traits. (XSLX 10 kb)

Additional file 7: Table S6a. Results of ImmunoChip fine-mapping by GUESSFM. (GZ 2833 kb)

Additional file 8: Table S6b. Results of GWAS summary statistic fine-mapping. (GZ 2833 kb)

Additional file 9: Table S7a. Autoimmune disease COGS gene prioritisation. Overall COGS gene scores (COGS_Overall_Gene_Score) for each gene and autoimmune disease are shown together with the prioritised category and score associated with that category (COGS_Category_Gene_Score) (Fig. 3). The ‘analysis’ column describes whether the input data was GWAS or ImmunoChip (ICHIP) and whether summary statistic (SS) or GUESSFM (GF) fine-mapping was used. ‘diff.expr’ indicates whether the gene was not expressed (NA) or, if expressed, whether there was differential expression at the FDR < 0.01 level (up, down or nsig). Similarly, ‘diff.erna’ indicates whether the HindIII fragment containing the strongest SNP signal is differentially expressed with the same categories. Using data from ImmunoBase (https://www.immunobase.org - accessed 06/06/2016), we annotate genes near (within 5 Mb) previously reported disease susceptibility regions, with contextual annotation ‘Closest_Disease_Susceptibility_Region’, ‘Closest_Min_P_Value_Susceptibility_SNP’, ‘Closest_Min_P_Value_Susceptibility_SNP_P_Value’, ‘PIR_Overlaps_Disease_DSR’ indicates that the PIR driving the prioritisation for a gene/disease overlaps an ImmunoBase known disease susceptibility region for that trait. Restricted to the subset of genes with scores > 0.5 that are analysed in this paper. (GZ 37 kb)

Additional file 10: Table S7b. As above, complete results. (GZ 37 kb)

Additional file 11: Table S8a. GTF file with definitions for all Ensembl 75 genomic features plus CD4-specific regulatory regions inferred from chromatin states. These regulatory regions have been named with identifiers containing a CD4R prefix, assigned a regulatory biotype and marked as pertaining to both genomic strands due to their bi-directional transcription potential. (GZ 39807 kb)

Additional file 12: Table S8b. Whole-genome segmentation of non-activated and activated CD4 T cells into 15 states obtained from a CHROMHMM analysis using ChIP-seq data for activated CD4^+^ T cells. (GZ 1551 kb)

Additional file 13: Table S8c. Whole-genome segmentation of non-activated and activated CD4 T cells into 15 states obtained from a CHROMHMM analysis using ChIP-seq data for non-activated CD4^+^ T cells. (GZ 1520 kb)

Additional file 14: Table S9. Genotypes for donors in the IL2RA ASE experiment across SNP groups A, C, D, E, F. (XLSX 12 kb)

Additional file 15: Table S10. Read counts for each allele at the IL2RA ASE experiment. The column Expt denotes sample id; time, the timepoint (0, 120, 240 min); stim, the condition (genomic DNA, time0 cDNA, stimulated or unstimulated cells cDNA). (GZ 4 kb)
