# Supplementary material for: Chromosome contacts in activated T cells identify autoimmune disease candidate genes
Source: Genome Biol. 2017 Sep 4;18:165. doi: 10.1186/s13059-017-1285-0 (PMC5584004; doi:10.1186/s13059-017-1285-0)
Supplement: Supplementary file 2 — Figure S1. Comparison of longer and shorter CD4+ T cell activation timecourses. Figure S2. Summary distributions of interacting fragments. Figure S3. Validation of PCHi-C by ChIA-PET. Figure S4. Chromatin state profiles of interacting fragments. Figure S5. Relationship of gene expression to PIR number and mRNA half-life. Figure S6. Definition and quantification of regulatory RNAs. Figure S7. blockshifter calibration. Figure S8. MDN1 is prioritised for RA through ImmunoChip but not GWAS data. Figure S9. Gene prioritisation using COGS. Figure S10. Multiple genes on chromosome 1q32.1 (IL10, IL19, IL20, IL24, FCAMR/PIGR) are prioritised for T1D, CRO and UC. Figure S11. Histograms show the distribution of summed PIR length by gene in non-activated CD4+ T cells (top panel) and TAD length in naive CD4+ T cells. Figure S12. IRF8 and EMC8/COX4I1 on chromosome 16 are prioritised for RA and SLE. Figure S13. AHR on chromosome 7 is prioritised for RA in activated CD4+ T cells. Figure S14. Allelic imbalance in mRNA expression in individuals heterozygous for group A SNPs is confirmed with reporter SNP rs12244380 (IL2RA 3’ UTR). (PDF 4243 kb) [file 13059_2017_1285_MOESM2_ESM.pdf]

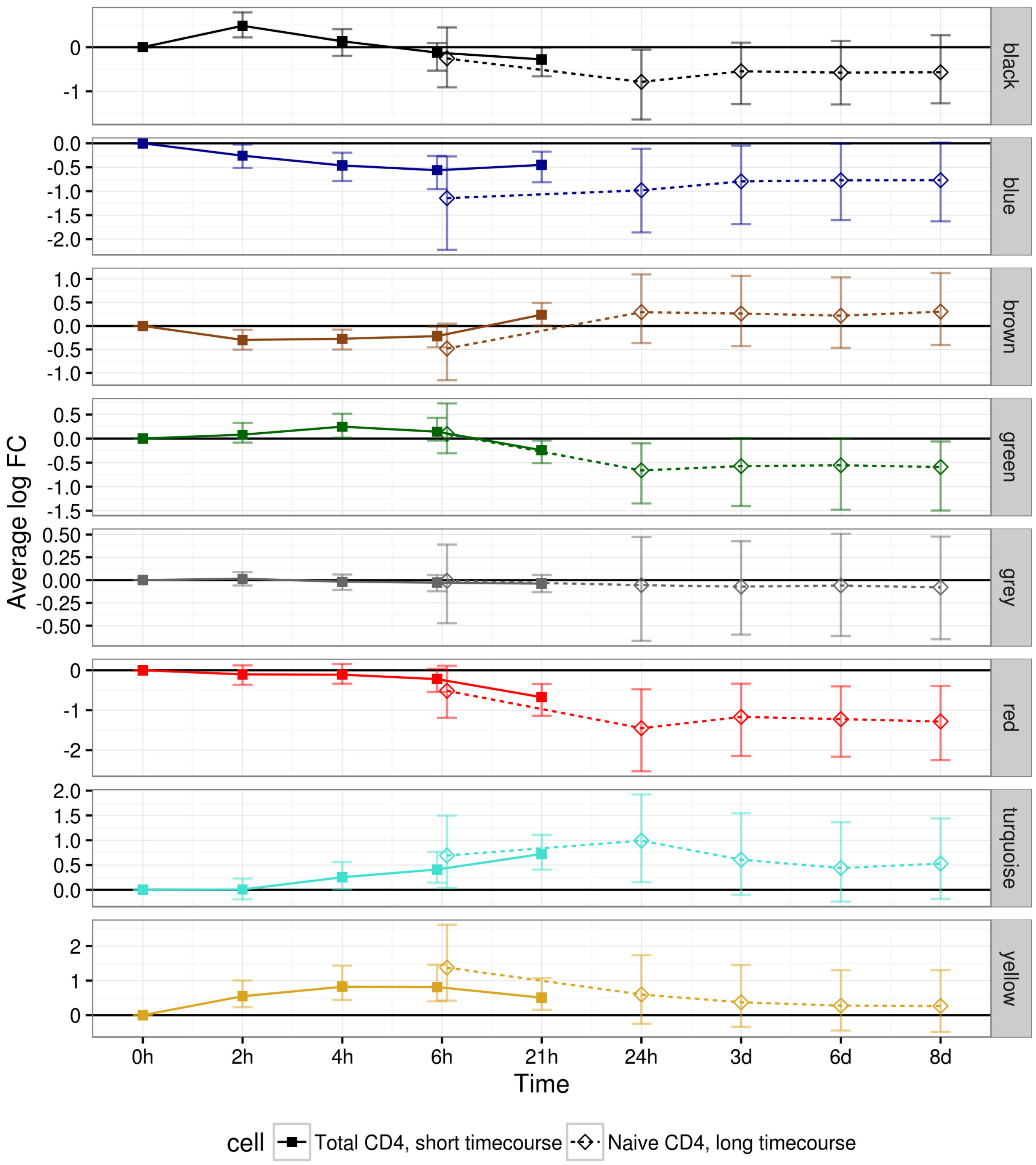

Fig. S1: **Comparison of longer and shorter CD4$^+$ T cell activation timecourses**. Microarray timecourse summary from this experiment (solid points) overplotted with a longer timecourse from GSE60680 (open points, Gustafsson et al, 2015). Points show the median log fold change amongst genes assigned to each module at each timepoint, with the interquartile range displayed as vertical ranges around each point. The results at 6 hours are slightly horizontally offset to allow the results from the two experiments to be visually distinguished. Note the non-linear mapping of time to the x axis, which contains a mixture of hours (h) and days (d), to allow visualization of the early timepoints in particular.

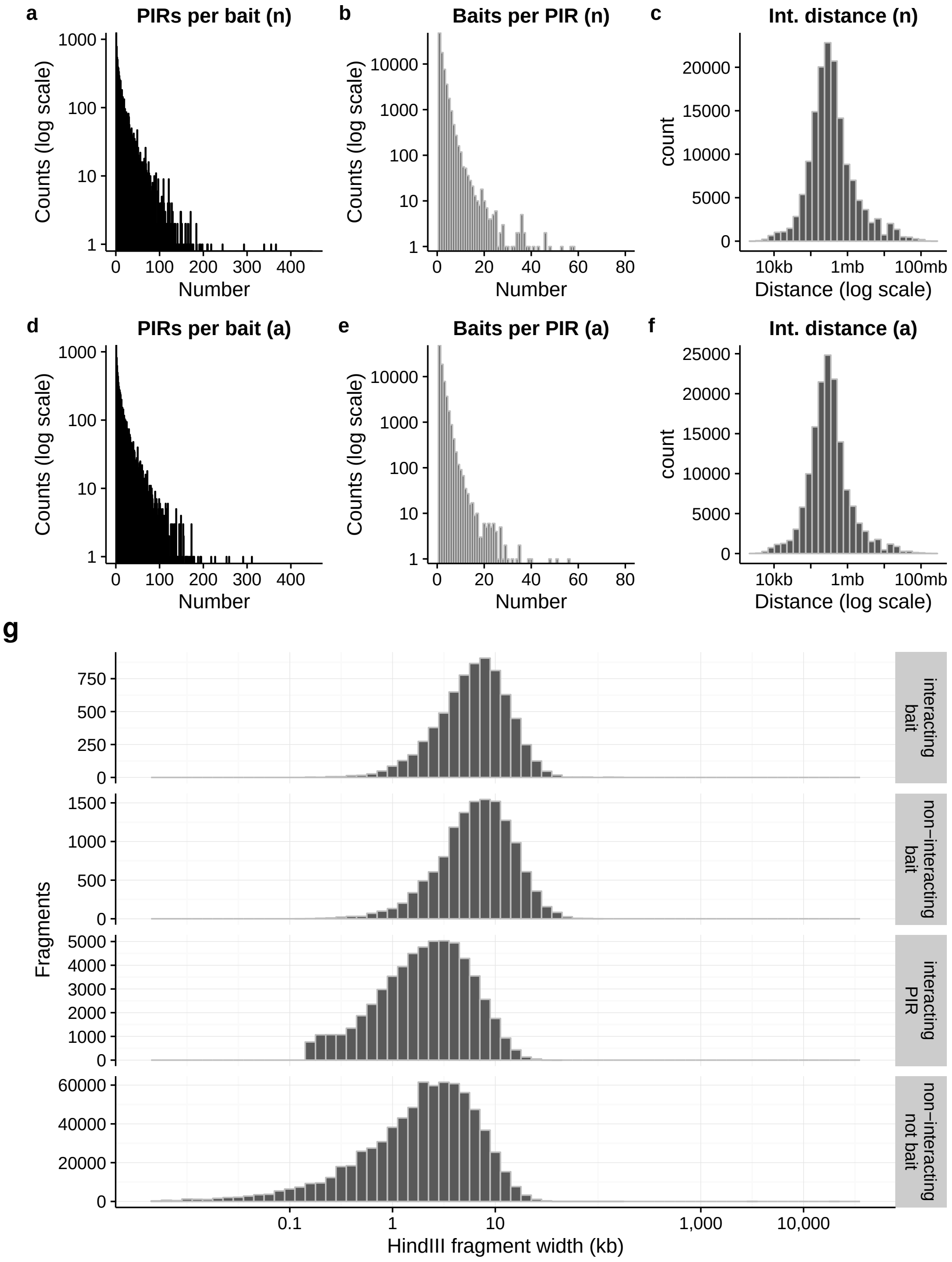

Fig. S2: **Summary distributions of interacting fragments.** Distributions of **a**, **d** number of interacting promoter bait fragments per PIR; **b**, **e** PIRs per promoter fragment; and **c**, **f** distance between midpoints of promoter and PIR *Hind*III fragments in activated (**a-c**) and non-activated (**d-f**) CD4^{+} T cells. **f** Width profile of *Hind*III fragments according to whether they were baited promoter fragments or not, and interacting fragments or not. **g** *Hind*III fragment length in the four categories of interacting and non-interacting baited fragments and PIRs.

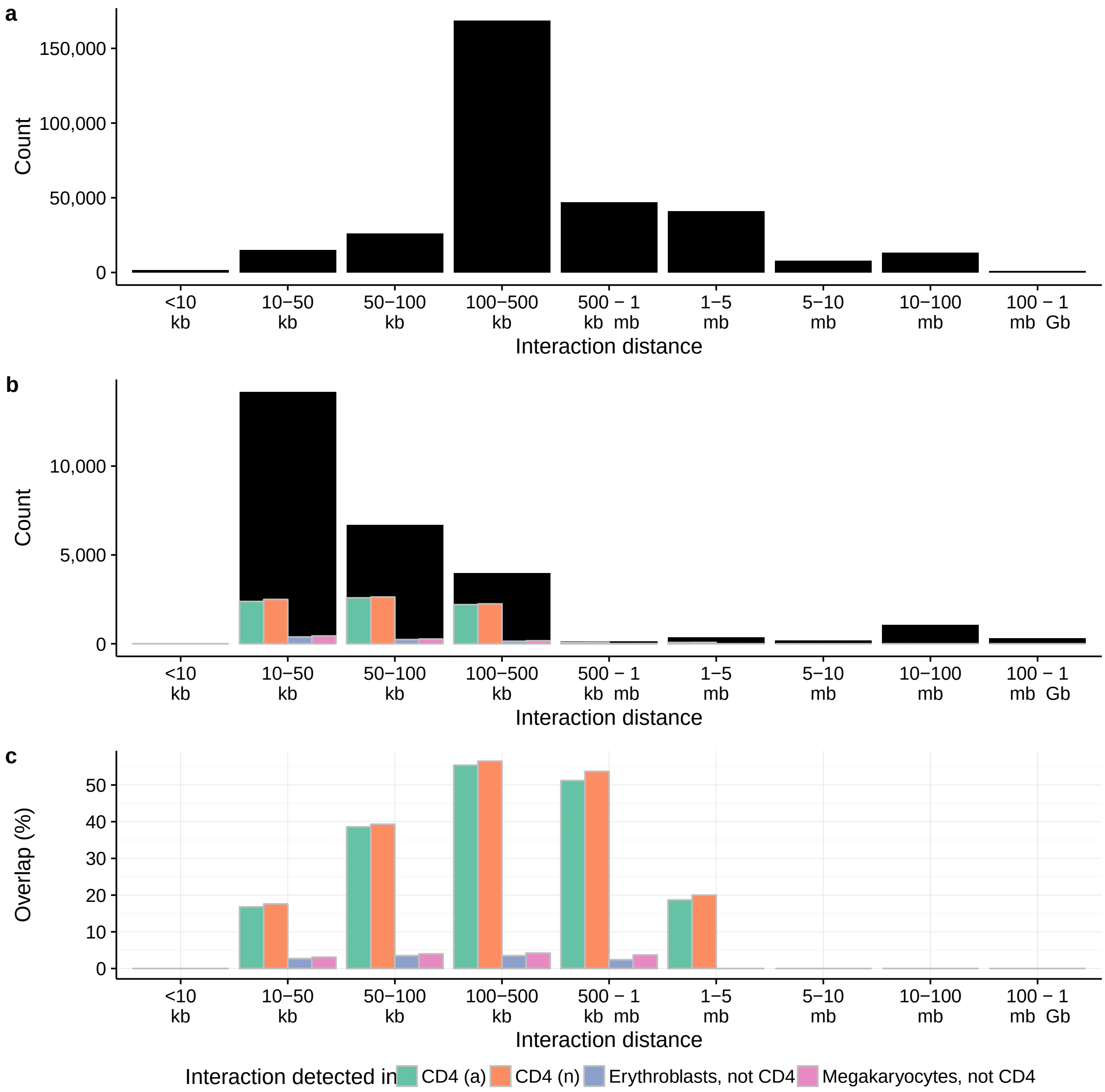

Fig. S3: **Validation of PCHi-C by ChIA-PET**. Distance profiles of PCHi-C and ChIA-PET derived promoter-enhancer interactions in **a** PCHi-C, non-activated $CD4^+$ T cells and **b** ChIA-PET (Chepelev et al), black bars. Coloured bars show the count (**b**) or percentage (**c**) of ChIA-PET interactions recovered in the PCHi-C experiment in non-activated and activated $CD4^+$ T cells (CD4 (a) and CD4 (n), respectively) and, for comparison, two non-lymphocyte cells, ethryroblasts and megakaryocytes processed in parallel after exclusion of interactions found in either $CD4^+$ T cell. Calling interactions requires correction for the expected higher density of random collisions at shorter distances[57] which are explicitly modelled by CHICAGO[9] used in this study but not in the ChIA-PET study[12]. As a result, we expected a higher false positive rate from the ChIA-PET data at shorter distances. Indeed, while we replicated only 17% of interactions in the 10-50kb range, we replicated over 50% of the longer range interactions (>100 kb).

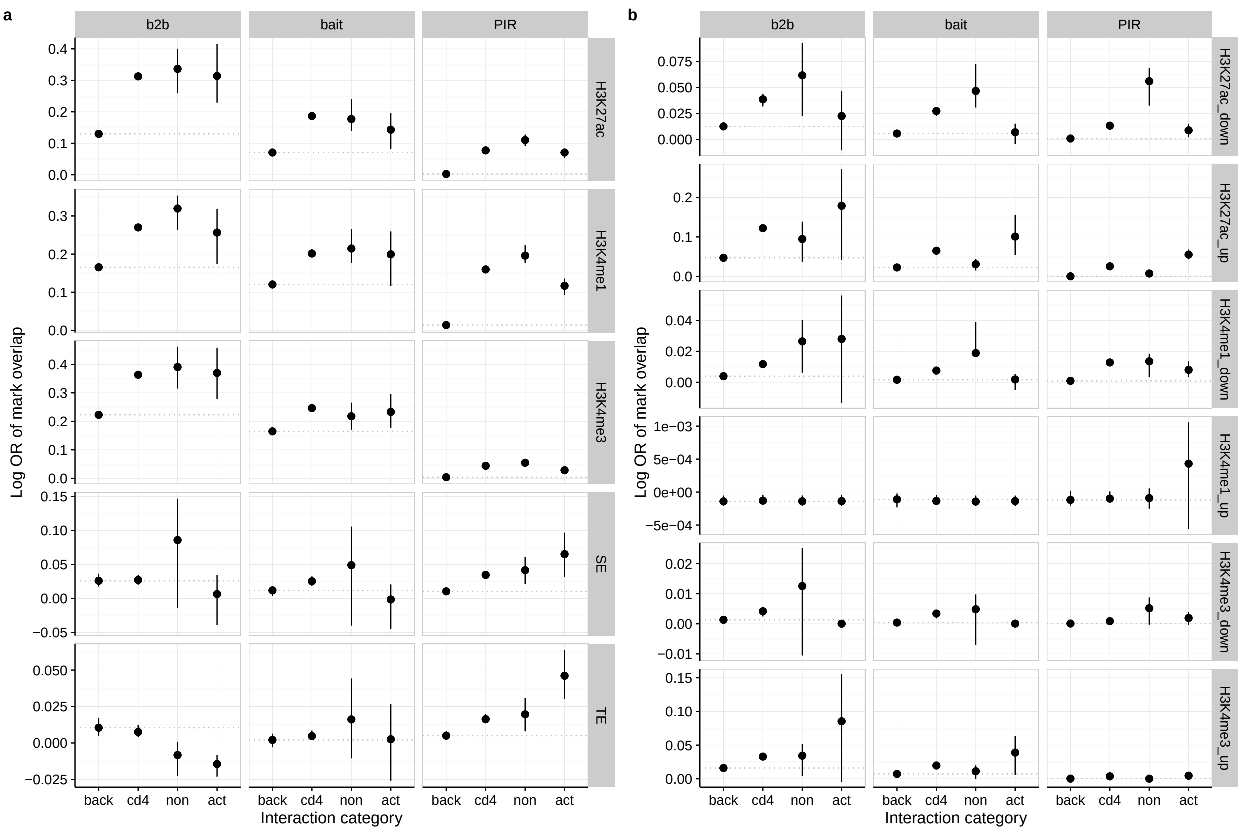

Fig. S4: **Chromatin state profiles of interacting fragments.** Log odds ratio that bait, bait-to-bait (b2b) and PIR regions detected in background cells (back; megakayrocytes and erythroblasts), activated and non-activated $CD4^+$ T cells (cd4), specifically non-activated or activated $CD4^+$ T cells (non or act, respectively) overlap (**a**) given ChIP-seq peaks or typical (TE) or super (SE) enhancers in resting T cells as previously defined[12] and (**b**) differential (FDR$<$0.1) ChIP-seq peaks compared to non-interacting regions. Regions considered specific to activated or non-activated cells had a CHICAGO score $>$ 5 only in that cell type and were considered differential interactions in a comparative analysis of mapped sequence counts at FDR$<$0.1.

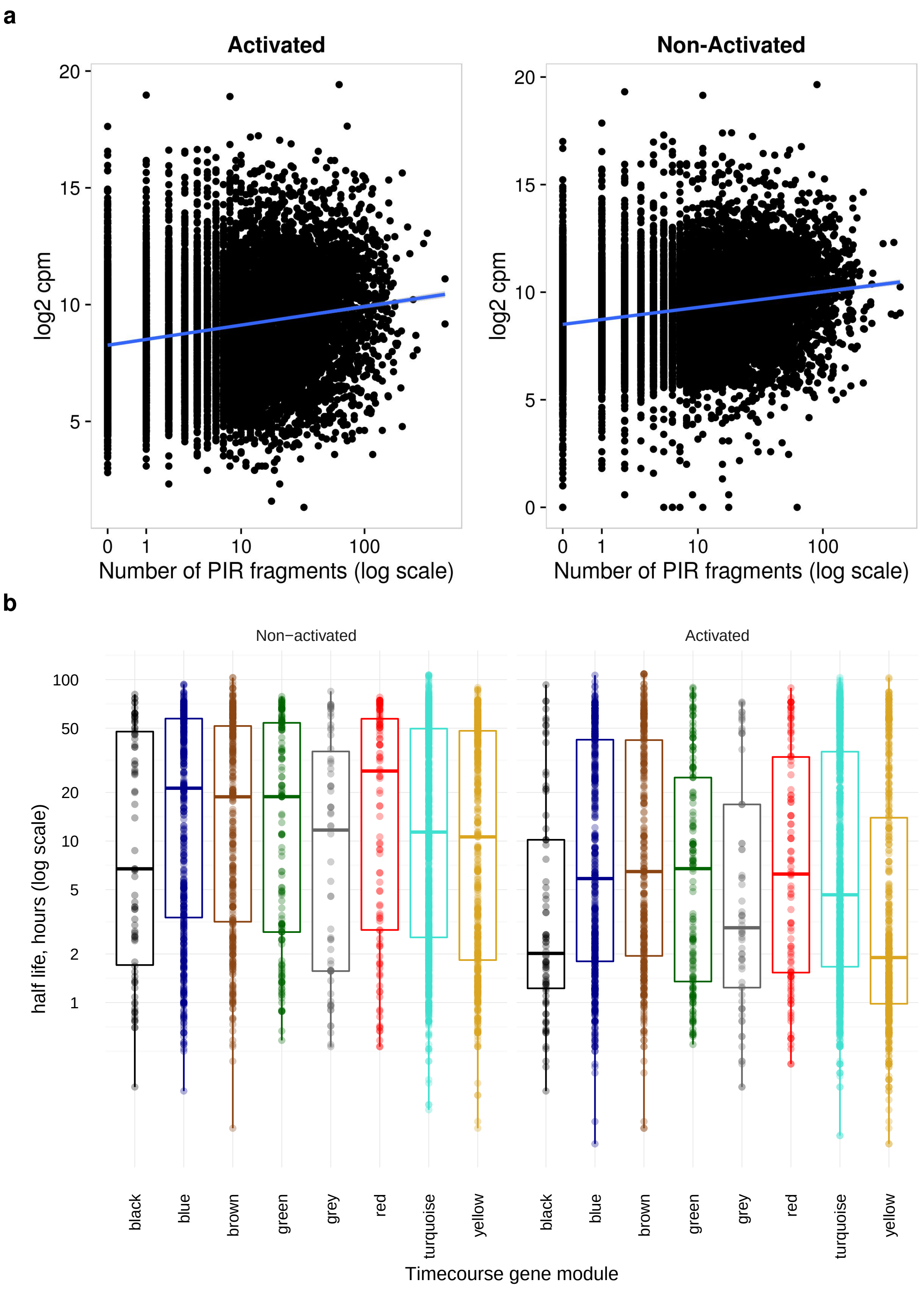

Fig. S5: **Relationship of gene expression to PIR number and mRNA half-life.** **a** RNA-seq expression (counts per million mapped reads, $\log_2$ scale) shows a positive correlation with the number of PIRs indentified through PCHi-C. **b** half-life of mRNA (Raghavan et al. 2002) by gene module in non-activated and activated cells. The most dynamically regulated genes in our time-course, those in the black module, had the shortest half-life ($p = 3 \times 10^{-8}$).

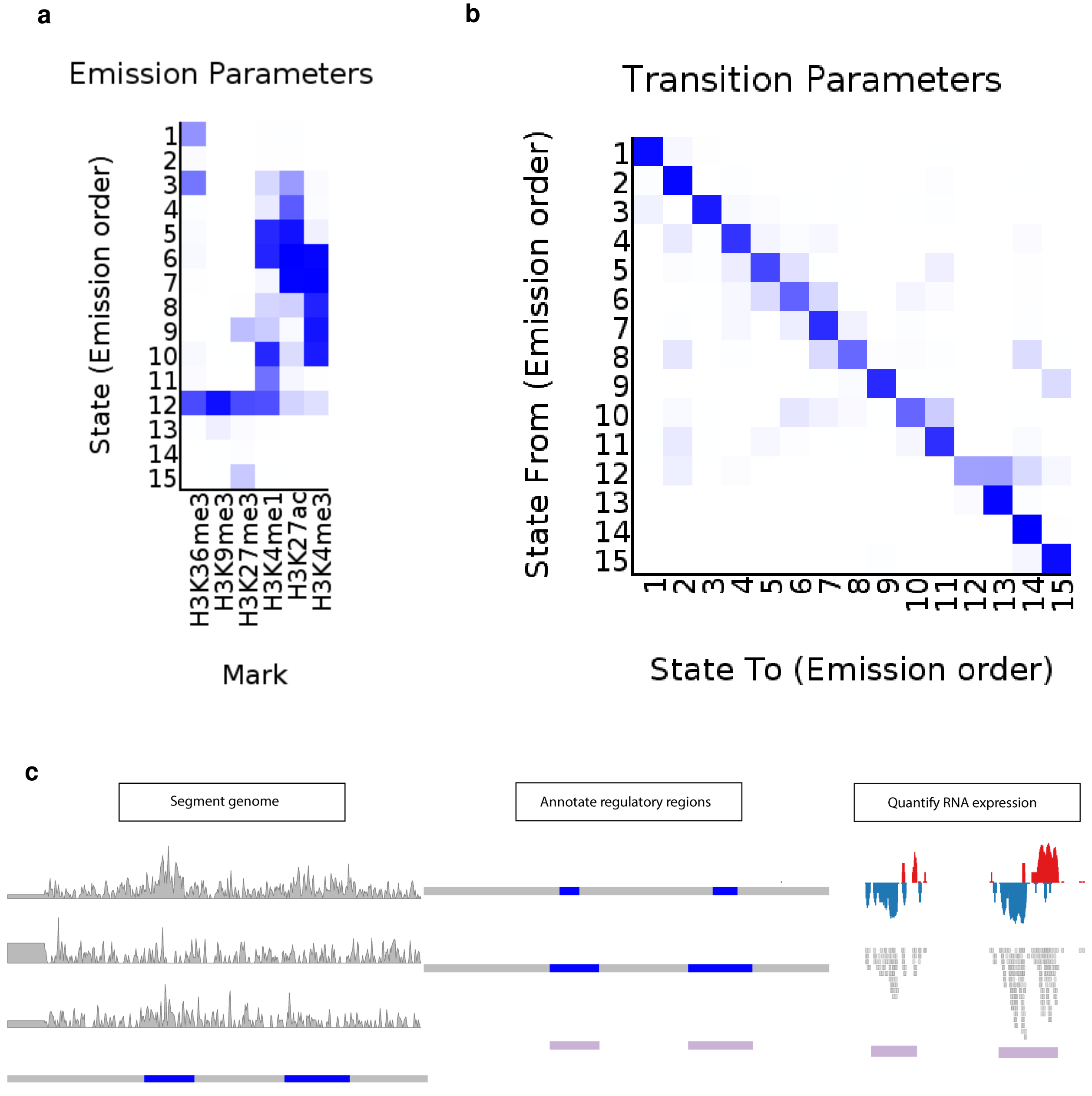

Fig. S6: Definition and quantification of regulatory RNAs. CHROMHMM analysis of ChIP-seq marks was used to produce a whole genome segmentation into 15 states. Resulting emission (**a**) and transmission (**b**) matrices are shown. States E4-E11 were defined as regulatory. **c** Neighbouring regions containing promoter or enhancer states (E4-E11) were merged together into regulatory annotations. Expression levels of each regulatory area were quantified using RNA-seq in a strand-aware fashion, to avoid the confounding effect of overlapping genomic features.

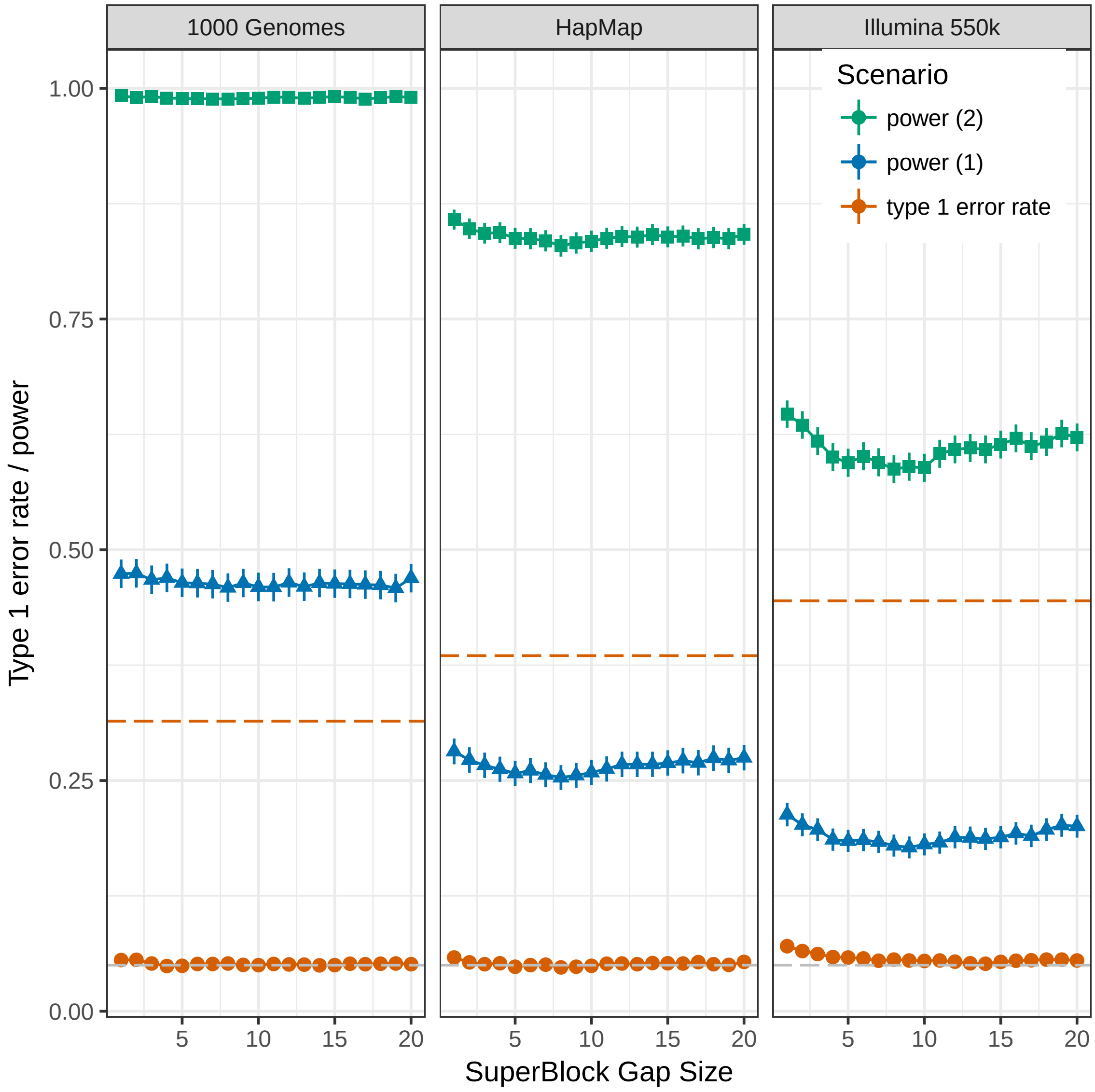

Fig. S7: blockshifter calibration. Each panel represents a simulated genotyping density: 1000 genomes (156,082 SNPs); HapMap (44,647 SNPs input,); Ilumina 550k (10,241 SNPs input). Points represent type 1 error rates (alpha=0.05) for the null scenario (no enrichment of GWAS variants in test specific PIRs) and moderate (power 1) and strong (power 2) enrichment scenarios across 4000 simulated GWAS, with differing blockshifter 'SuperBlock' gap size parameter. Error bars represent 95% confidence intervals. Dashed red lines represent the type 1 error rate for Fisher's test of enrichment of variants in test and control PIRs. The naive application of Fisher's test leads to substantial inflation of type 1 error rate, more so in lower-density genotyping scenarios. Blockshifter maintains type 1 error rate control, although a gap size of 5 or more is required to deal with the extended correlation induced by PMI in lower density genotyping scenarios, while Blockshifter power is impacted, as expected, by genotyping density.

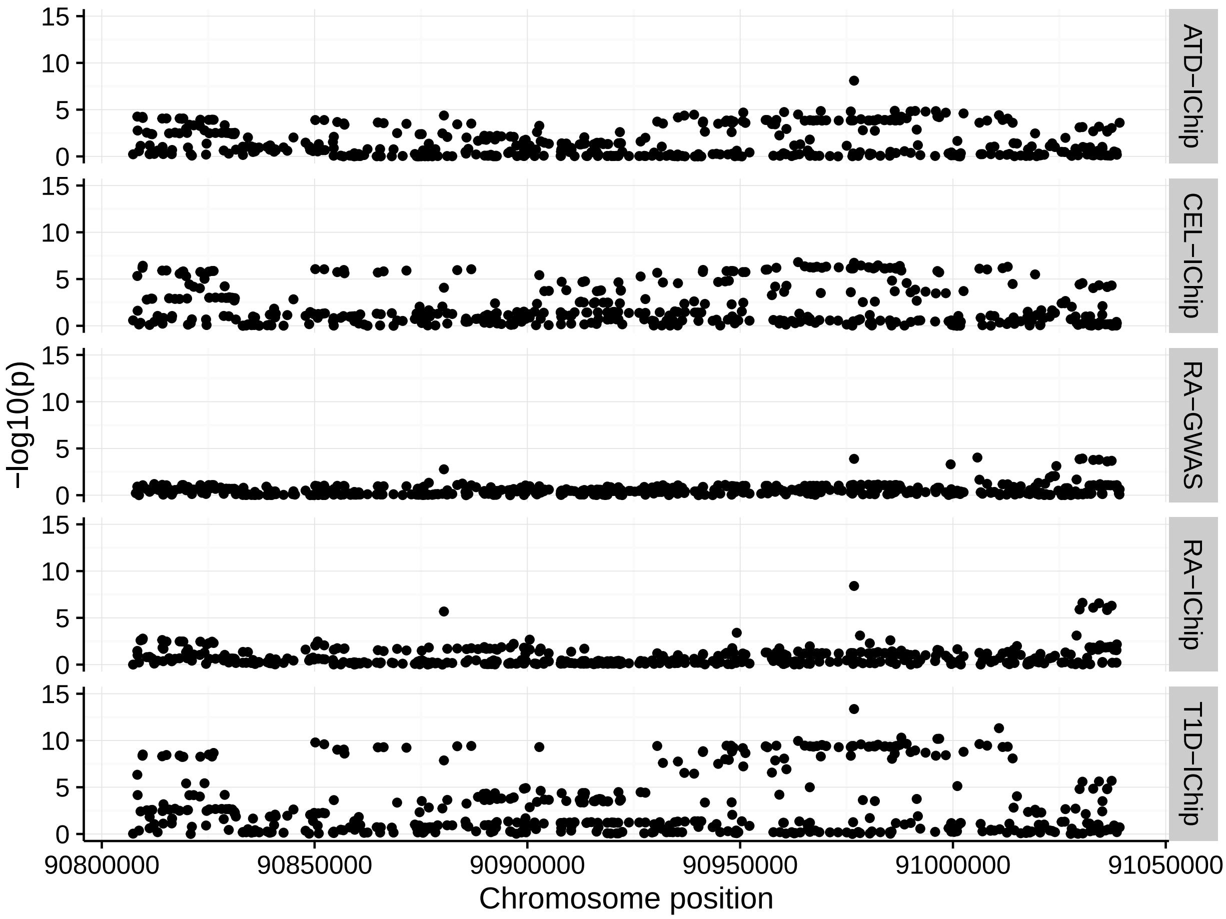

Fig. S8: *MDN1* is prioritised for RA through ImmunoChip but not GWAS data. Similar signals are found for ATD and T1D, which also link to *MDN1*, supporting the RA-ImmunoChip result. The lack of prioritisation in the RA-GWAS dataset relates to the weaker evidence for association in this region.

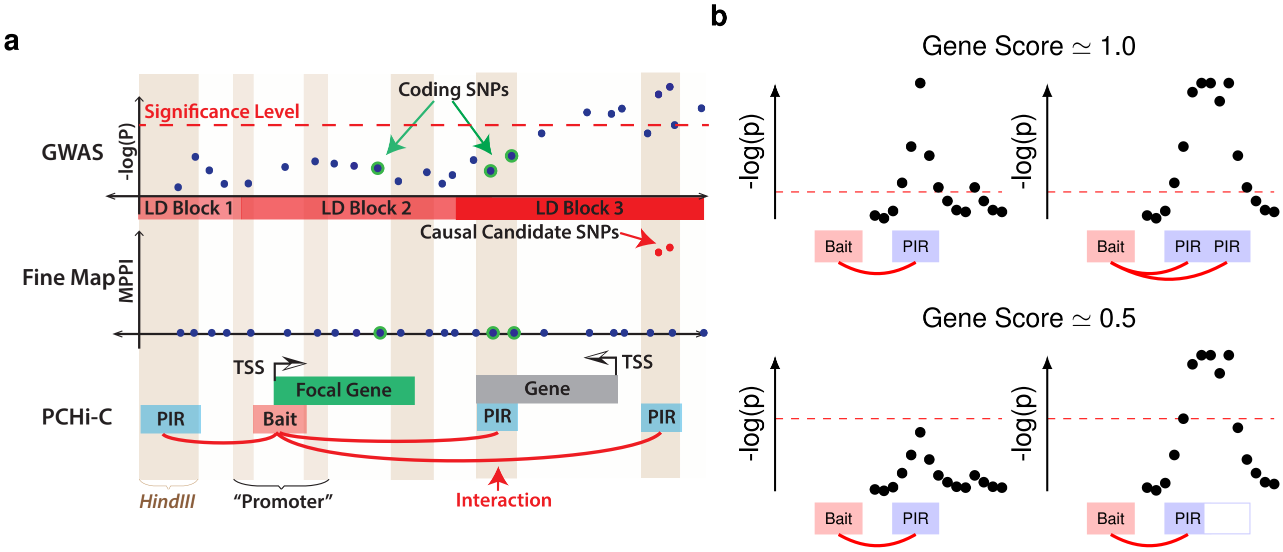

Fig. S9: **Gene prioritisation using COGS.** We prioritised disease candidate causal genes by integrating GWAS data with PCHi-C interactions using the COGS algorithm. **a** The algorithm uses a Bayesian method to define the marginal posterior probability of inclusion (MPPI, middle panel) for each variant from GWAS data (top panel). We can also calculate the MPPI marginalising across PIRs (light blue, bottom panel), coding variants and promoter regions for each focal gene. *Hind*III fragments are indicated by dark/light vertical shading. **b** Note that the gene score is therefore a function of the strength of GWAS signal, how peaked/diffuse it is, and the interactions. For example, in the top row there are two strong GWAS signals, one peaked, one diffuse, but the PIRs cover all of the most strongly associated SNPs, and in each case the gene score is expected to be close to 1. In the lower left plot, the GWAS signal is less strong, not even genomewide significant, but all the most associated SNPs lie within the PIR. The score will fall, perhaps to around 0.5, reflecting the weaker evidence for disease association. In contrast, the bottom left plot shows a diffuse signal, only part of which lies within a PIR. Although we can be confident the disease is genuinely associated, only about half the fine mapped candidate causal SNPs will lie within a PIR, and the gene score will again fall, to about 0.5. The situations in the lower row are quite different, but will generate similar scores.

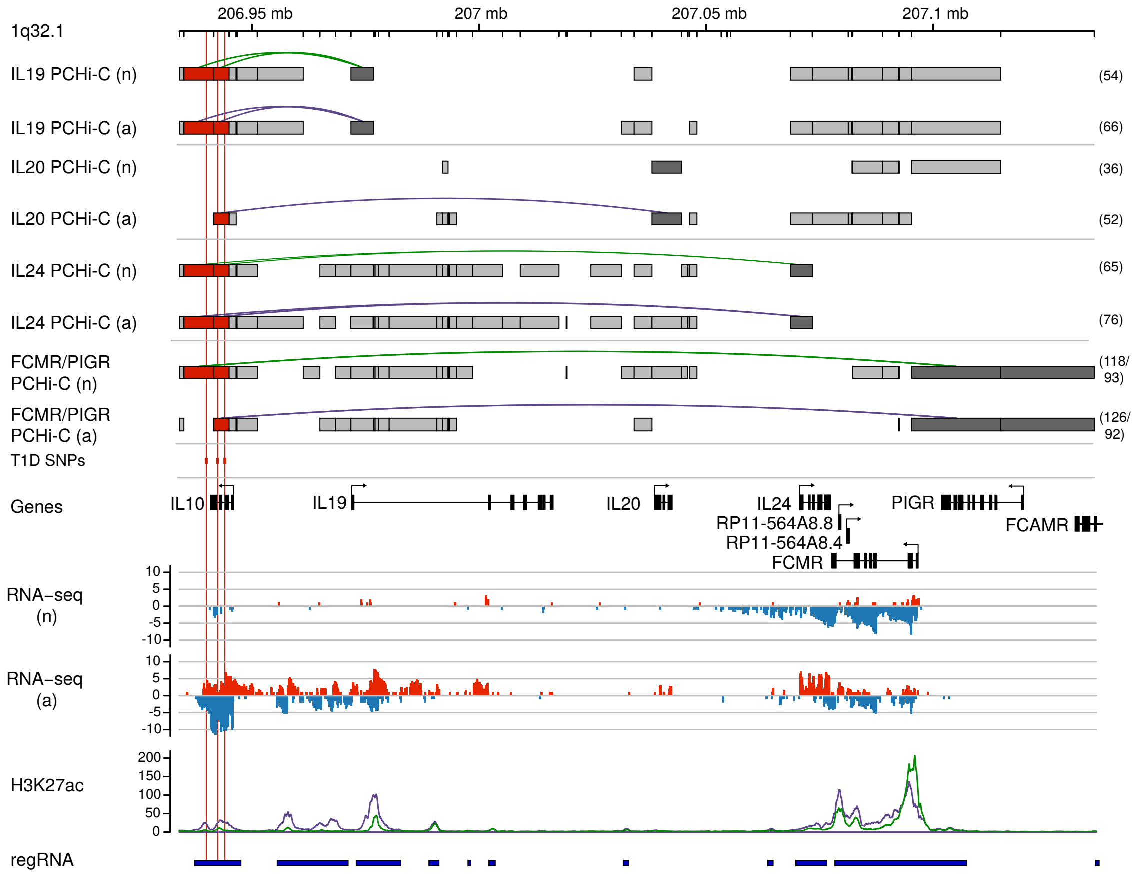

Fig. S10: Multiple genes on chromosome 1q32.1 (*IL10*, *IL19*, *IL20*, *IL24*, *FCAMR*/*PIGR*) are prioritised for T1D, CRO and UC. For full legend see **Fig. 5**.

.

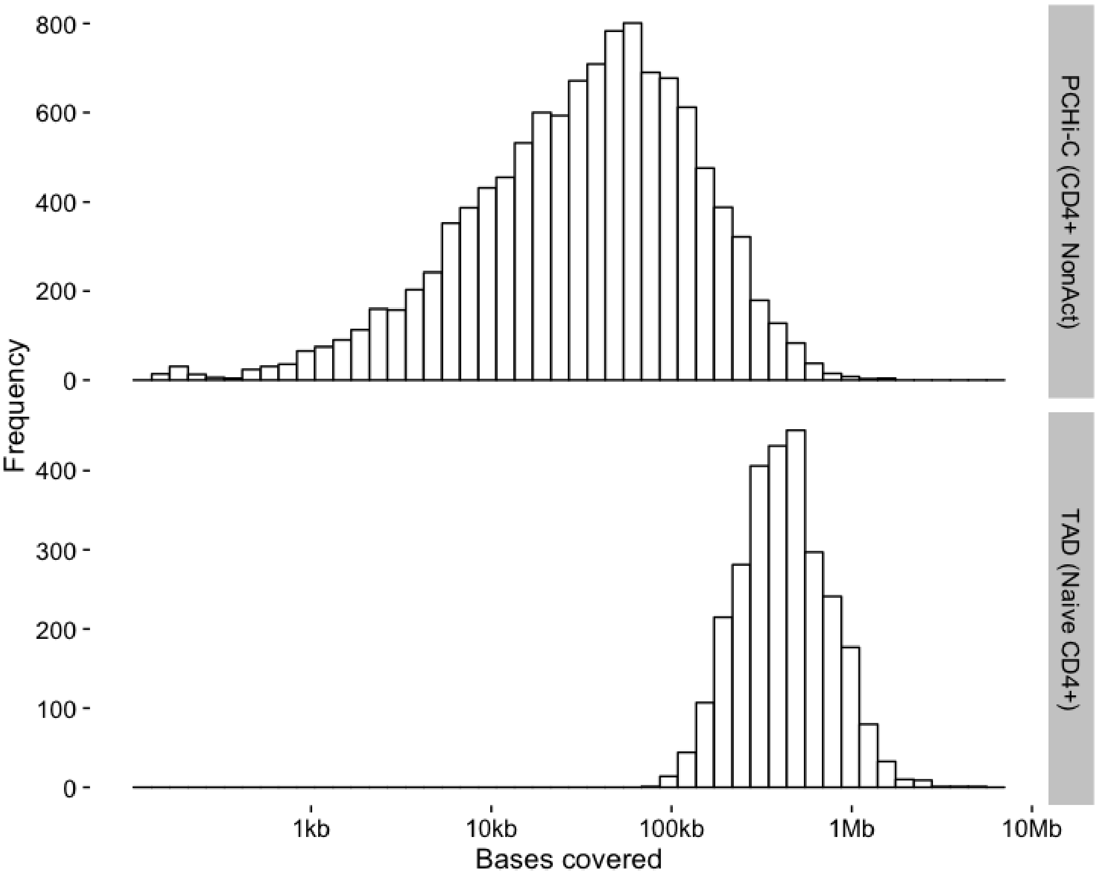

Fig. S11: Histograms show the distribution of summed PIR length by gene in non-activated $CD4^{+}$ T cells (top panel) and TAD length in naive $CD4^{+}$ T cells. Note the x axis is drawn using a log scale and that for each gene we have included the promoter-baited fragment and its two immediate neighbours to allow that PCHi-C cannot detect very proximal interactions in this range.

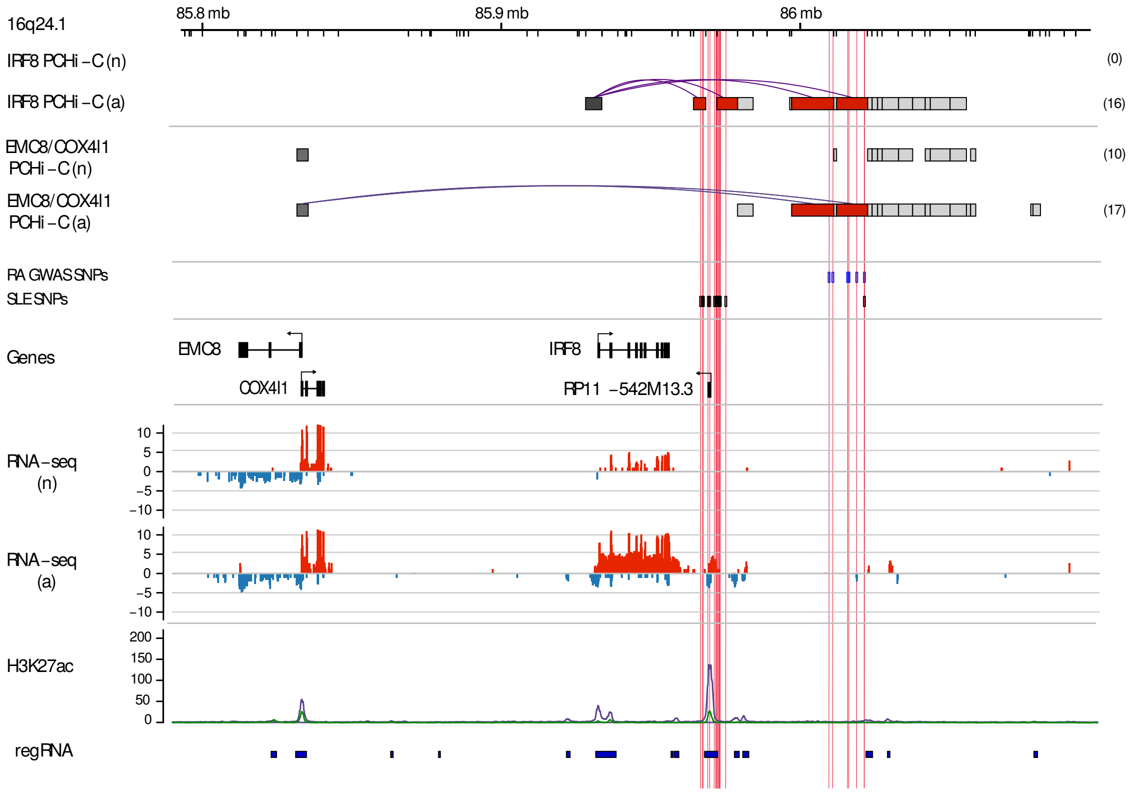

Fig. S12: *IRF8* and *EMC8*/*COX4I1* on chromosome 16 are prioritised for RA and SLE. For full legend see **Fig. 5**.

.

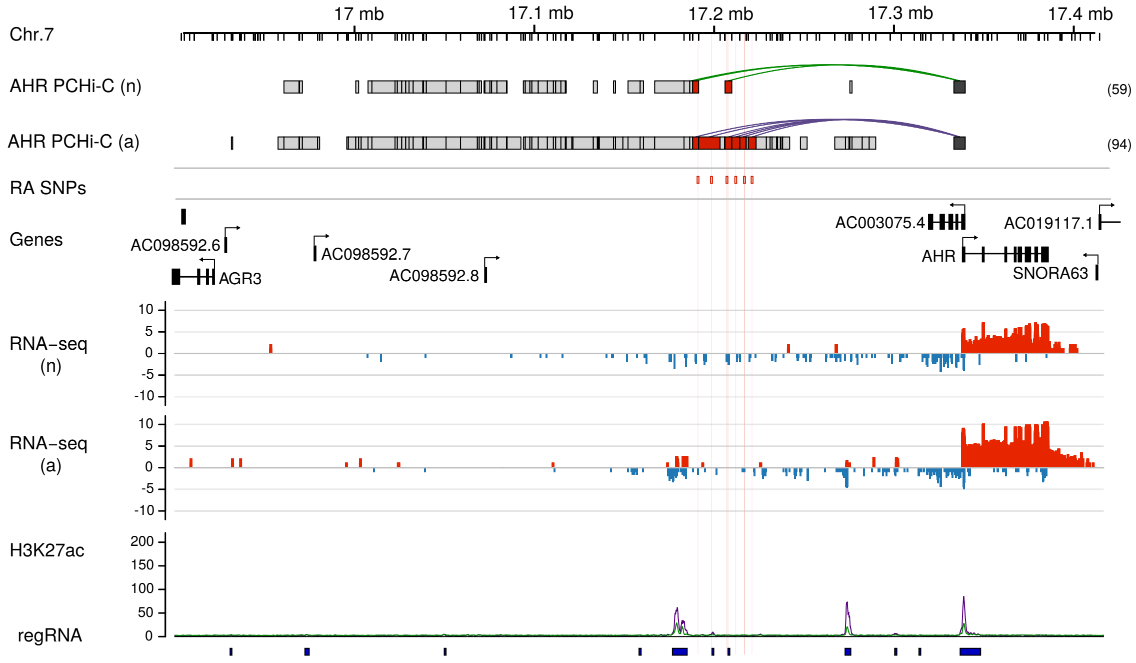

Fig. S13: *AHR* on chromosome 7 is prioritised for RA in activated $CD4^+$ T cells. For full legend see **Fig. 5**.

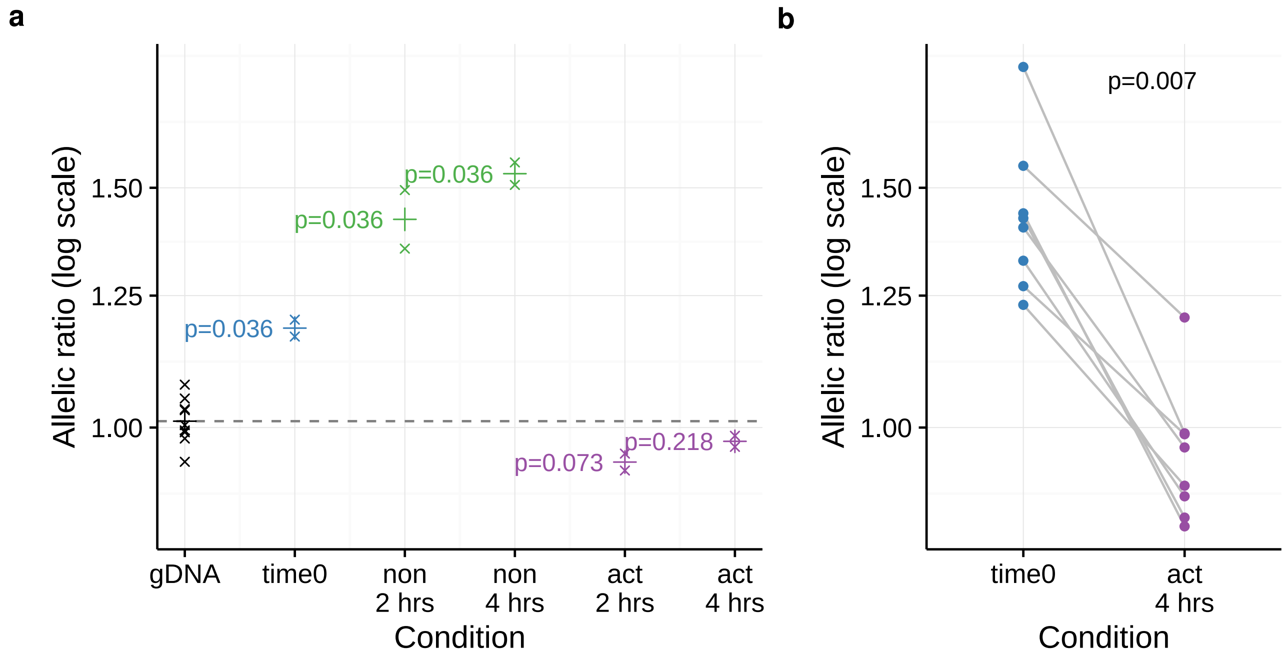

Fig. S14: **Allelic imbalance in mRNA expression in individuals heterozygous for group A SNPs is confirmed with reporter SNP rs12244380 (*IL2RA* 3' UTR)** **a**: Allelic imbalance in mRNA expression in total CD4$^+$ T cells from individuals heterozygous for group A SNPs using rs12244380 as a reporter SNP in non-activated (non) and activated (act) CD4$^+$ T cells compared to genomic DNA (gDNA, expected ratio=1). Allelic ratio is defined as the ratio of counts of the allele carried on the chromosome carrying rs12722495:T to that carried on the chromosome carrying rs12722495:C. '×'=geometric mean of the allelic ratio over 2-3 replicates within each of 4-5 individuals, and p values from a Wilcoxon rank sum test comparing cDNA to gDNA are shown. '+' shows the geometric mean allelic ratio over all individuals. **b**: Allelic imbalance in mRNA expression in memory CD4$^+$ T cells differs between *ex vivo* (time 0) and four hour activated samples from eight individuals heterozygous for group A SNPs using rs12244380 as a reporter SNP. p value from a paired Wilcoxon signed rank test is shown.
